# Computationally Evidence‐Grounded Sequence‐First Design of Peptide Binders

**DOI:** 10.1002/advs.77125

**Published:** 2026-08-14

**Authors:** Wenze Ding

**Affiliations:** ^1^ School of Life and Environmental Sciences University of Sydney New South Wales Australia; ^2^ School of Mathematics and Statistics University of Sydney Sydney New South Wales Australia; ^3^ Charles Perkins Center University of Sydney Sydney New South Wales Australia

**Keywords:** peptide design, protein‐peptide interactions, retrieval‐augmented generation, sequence‐first generation, topology‐conditioned decoding

## Abstract

Peptide binders provide a versatile modality for modulating protein targets that are poorly addressed by small molecules, but their discovery is constrained by sample‐intensive screening or reliance on structural templates. Sequence‐first generation offers a scalable alternative for targets lacking stable or representative structures, yet existing approaches often sacrifice target‐specific control for diversity and are further limited by the imperfect transfer of protein language‐model priors to short peptides. Here, we report BOND‐PEP, an evidence‐grounded framework for sequence‐only peptide binder generation. BOND‐PEP retrieves target‐relevant peptide exemplars, aligns them with the query protein through bipartite message passing, and uses the resulting protein‐centric representation to guide conditional decoding. In a matched AlphaFold‐Multimer evaluation on a non‐homologous held‐out benchmark, BOND‐PEP improved reference‐beating ipTM success over RFdiffusion, PepPrCLIP and PepMLM. It further transferred to a compact external panel of targets with previously reported experimentally supported peptide binders. These results establish retrieval‐augmented, topology‐conditioned decoding as a practical route to controllable peptide binder design.

## Graphical abstract

BOND‐PEP enables controllable, sequence‐first peptide binder design by grounding generation in binding evidence retrieved for each target. It uses topology‐conditioned message passing to integrate relevant peptide examples with the target protein sequence, forming a residue‐level representation that guides the generation of diverse, target‐specific binders for unseen proteins, without requiring an experimentally determined or predicted target structure.

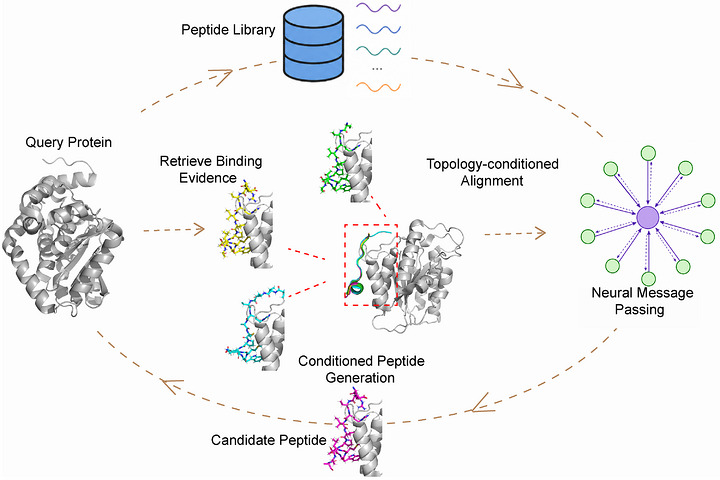

## 1 Introduction

Peptide binders provide a direct route to modulate proteins that are difficult to drug with small molecules, including targets lacking well‐defined pockets, exhibiting substantial conformational plasticity or containing intrinsically disordered regions [1, 2, 3, 4]. Notably, estimates suggest that upward of four in five disease‐associated proteins remain undruggable with conventional small‐molecule strategies, underscoring the scale of this unmet need [5, 6, 7, 8]. Their therapeutic potential spans competitive inhibition, induced proximity, and targeted degradation [9, 10, 11]. Yet the discovery of high‐affinity, selective peptides remains labor‐intensive and iterative [12, 13, 14, 15, 16, 17]. From a learning and generalization perspective, peptide binder design is a conditional generation problem under severe constraints: only the target protein is consistently available; the design space grows exponentially with peptide length; training labels are sparse and noisy; and practical success requires robustness to distribution shift across protein families, binding modes, and disorder content [2, 3, 18, 19, 20, 21, 22]. Bridging this gap calls for generative methods that are both expressive and controllable under realistic data imperfections.

A dominant tradition in peptide binder design is structure‐centric. Ranging from docking and Rosetta‐style optimization to modern structure‐conditioned neural generators, these approaches can directly enforce geometric complementarity and physicochemical constraints when reliable three‐dimensional information is available [18, 19, 22, 23, 24, 25, 26, 27, 28, 29]. However, structure‐driven workflows inherit several practical bottlenecks: many targets lack high‐confidence structures in relevant conformational states; binding often involves flexible or intrinsically disordered regions where a single static template is inadequate; errors in structure prediction or docking can be amplified during design; and the resulting multi‐stage pipelines can be computationally heavy and brittle. These limitations motivate complementary sequence‐first approaches that can operate when structure is missing, uncertain, or conformationally heterogeneous.

In parallel, large protein language models have enabled sequence‐based representations with coevolutionary dependencies and residue‐level constraints [30, 31, 32, 33, 34, 35]. Embeddings with such signals provide a practical substrate for peptide binder discovery and design without explicit structural templates. However, whether protein‐trained PLMs transfer uniformly across sequence regimes remains unclear.

Recent sequence‐first methods fall broadly into two paradigms [2, 3, 36, 37, 38, 39]. The first follows a “generate‐then‐rank” loop: a generator proposes many candidate peptides, and a contrastively learned compatibility model ranks and selects those most consistent with the target. This decoupling offers flexibility and can exploit large collections of unlabeled sequences, but it also creates unstable search dynamics, where success depends on extensive sampling and strong post hoc filtering. The second paradigm performs direct target‐conditioned generation by casting peptide design as conditional sequence infilling, where a masked binder span is reconstructed end‐to‐end from the target sequence. While this can improve coherence and reduce reliance on downstream filtering, the conditioning is frequently implicit, being encoded as general context rather than as explicit residue‐level evidence of target‐specific preferences.

Existing sequence‐first pipelines therefore still struggle with the central tension between creativity and controllability, namely exploring novel sequences while remaining anchored to actionable binding evidence. What remains missing is a principled bridge from evidence to generation that simultaneously (i) grounds peptide priors in peptide‐relevant statistics rather than assuming protein‐derived priors transfer, and (ii) aligns target residues with candidate peptide patterns and exposes the resulting preferences as explicit conditioning.

**FIGURE 1:**
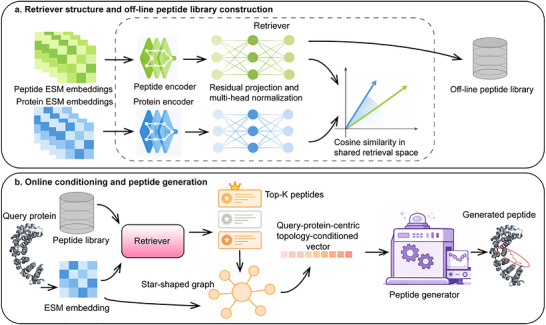
Target‐conditioned peptide retrieval and topology‐conditioned peptide generation. (a) Retriever architecture and off‐line peptide library construction. ESM‐derived embeddings of peptides and proteins are fed into peptide and protein encoders, respectively, followed by a residual projection module with multi‐head normalization to map both modalities into a shared retrieval space. Candidate peptides are then ranked by cosine similarity between the query protein and peptide representations. Peptide embeddings for the entire library are precomputed and indexed off‐line to enable fast search at query time. (b) Online conditioning and peptide generation. Given a query protein, its embedding is used to retrieve the top peptides from the pre‐prepared indexed library. These retrieved peptides are used to construct a protein‐centerd star graph for message passing. A query‐centric, topology‐conditioned vector is then derived and provided as conditioning input to a peptide generator to produce new peptides. The binding pose of the generated peptide to the query protein is highlighted at right with a red ellipse.

Here we address this gap by introducing BOND‐PEP, an evidence‐grounded framework for sequence‐first peptide binder generation. BOND stands for “Bipartite cONditioned Decoding”, and “evidence‐grounded” here means BOND‐PEP couples target‐conditioned peptide retrieval with topology‐conditioned bipartite alignment to convert empirical binding evidence into an explicit, residue‐resolved conditioning state for generation (Figure 1). A small set of target‐relevant peptide candidates is first retrieved to anchor decoding in a locally plausible region of sequence space. Their residue‐level correspondences with the query protein are then integrated through protein‐peptide message passing to produce a protein‐centric representation that guides conditional decoding. Under a matched AlphaFold‐Multimer‐based evaluation on a non‐homologous held‐out benchmark, BOND‐PEP achieved 65.8% reference‐beating ipTM success, exceeding RFdiffusion, PepPrCLIP and PepMLM, and it further transferred to a compact external panel of targets with previously reported experimentally supported peptide binders. These results establish BOND‐PEP as a computational framework for controllable peptide binder generation in sequence‐only settings.

## 2 Results

### 2.1 Protein Language Models Fail to Transfer Uniformly to Peptide‐length Regimes

Protein language models (PLMs) that learn transferable representations on large‐scale protein sequences have become the most widely used foundation models for protein‐sequence‐related analysis and tasks. However, their performance in the short, limited‐context sequence regime where peptide binder design operates has not been systematically evaluated. Here, we selected two widely adopted and state‐of‐the‐art PLMs, ESM‐2 [31] and more recent ESM‐C [32], and compared them on randomly sampled 1000 proteins and 1000 peptides. We evaluated these two models on three complementary sequence‐modelling tasks: (i) self‐copy (sequence self‐consistency when the full sequence is provided), (ii) LOO (leave‐one‐out masked recovery, i.e., predicting one held‐out residue at a time), and (iii) denoising (iterative denoising from partially corrupted inputs). For visualization and summary within the peptide evaluation set, the 1000 peptides were grouped into ≤ 10, 11–15, and > 15 amino‐acid bins, making them an interpretable and reasonably balanced presentation scheme.

**FIGURE 2:**
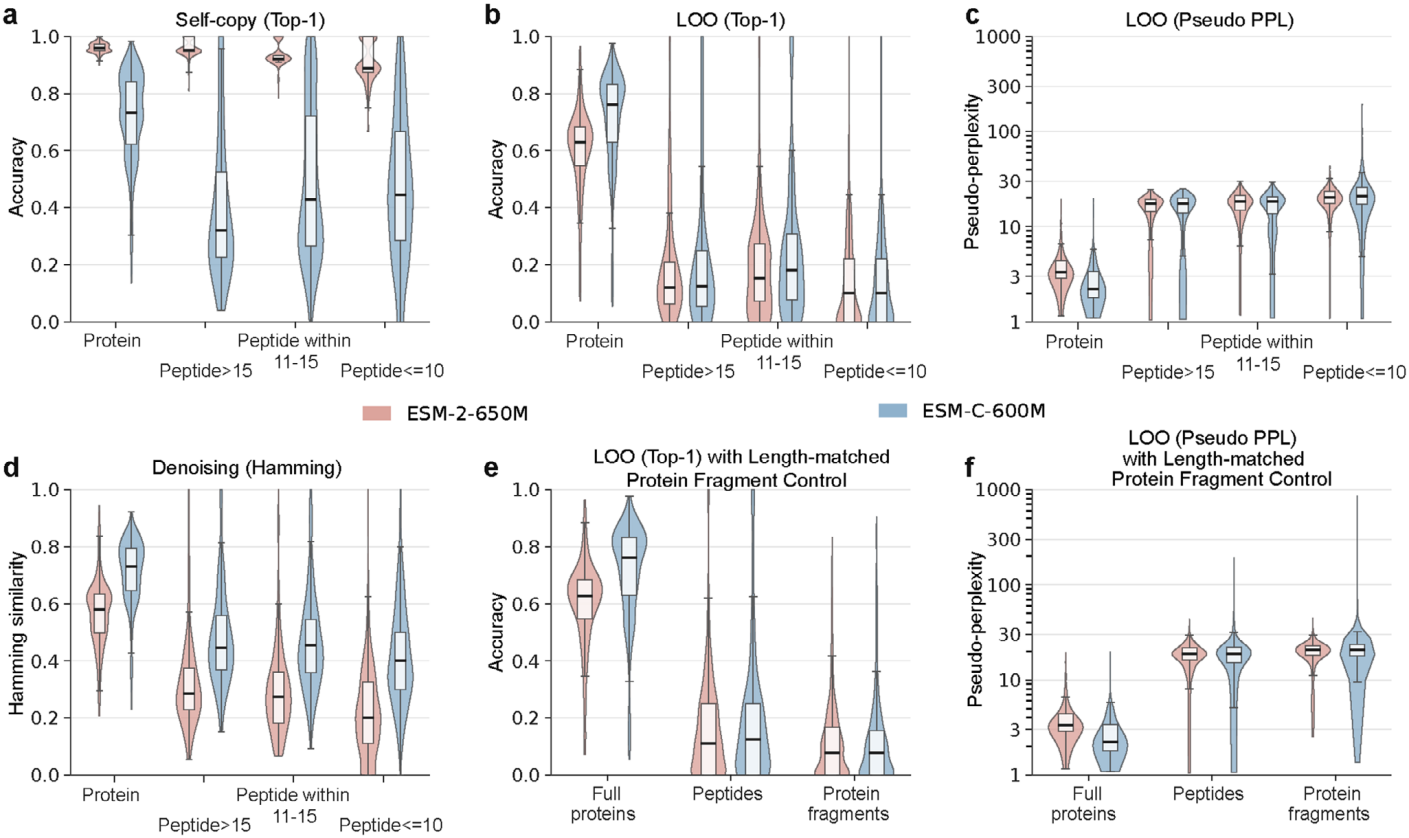
Protein language models transfer unevenly to peptide‐length sequence regimes. We compared ESM‐2‐650 M (pink) and ESM‐C‐600 M (blue) on proteins, peptides and length‐matched protein fragments. For each category, 1000 sequences were randomly sampled from our sequence dataset. (a) Self‐copy (Top‐1). Top‐1 accuracy when the full sequence is provided as input and the model is evaluated on recovering the same residues. (b) Leave‐one‐out (LOO; Top‐1). Top‐1 accuracy when one residue is masked at a time and predicted from the remaining context. (c) LOO (pseudo perplexity). Predictive uncertainty under LOO, computed from per‐position negative log‐likelihood and exponentiated. Lower values indicate higher confidence. The y axis is log scale. (d) Denoising (Hamming similarity). Reconstruction fidelity after iterative denoising from partially corrupted inputs, quantified as the fraction of positions matching the ground‐truth sequence. (e, f) Protein fragments were sampled from full‐length proteins to match the peptide length distribution and evaluated with the same PLM scoring pipeline. Violin plots show the distribution across sequences, with embedded boxplot summaries.

As shown in Figure 2, we found satisfactory performance on proteins but a pronounced degradation on peptides. Across tasks, proteins consistently formed a “high‐performance regime,” whereas peptides, especially those ≤ 10 amino acids, showed a sharp drop in recoverability and confidence. Meanwhile, these two PLMs showed complementary strengths. ESM‐2 consistently outperformed ESM‐C in the self‐copy task. By contrast, ESM‐C generally matched or exceeded ESM‐2 when the input was partially observed.

More specifically, in the self‐copy task, ESM‐2 approached near‐saturated Top‐1 accuracy across all regimes, yet performance degraded on peptides, with the clearest decline for short peptides. In parallel, ESM‐C remained strong on proteins but showed lower self‐copy Top‐1 recovery on peptides, centered around 0.4 accuracy, across peptide length bins (Figure 2a). The protein‐peptide gap was more pronounced in the LOO task, which probes whether a model can infer a residue from its surrounding context rather than simply echoing it. Both models achieved markedly higher Top‐1 LOO accuracy on proteins but performed poorly on peptides (Figure 2b). Consistently, the pseudo‐perplexity of the two models was lowest for proteins and substantially higher for peptides, with the shortest peptides showing the highest uncertainty (Figure 2c). Finally, in the denoising task, where sequences were corrupted and iteratively reconstructed, both models again showed a clear protein–peptide gap. Compared with proteins, which were recovered with higher Hamming similarity, peptides exhibited reduced reconstruction fidelity, and the deficit increased with decreasing peptide length (Figure 2d).

To investigate whether the observed degradation was primarily associated with peptide identity or with reduced sequence context in short sequences, we further generated length‐matched protein fragments from full‐length proteins and evaluated them with the same PLM scoring pipeline (Figure 2e,f). The fragment control largely reproduced the reduced recoverability and elevated uncertainty observed before, indicating that short‐sequence context contributes substantially to this pattern. Independently, to ensure that the peptide‐length trend was not an artifact of the original ≤ 10, 11–15, and > 15 length bins, we added three cutoff‐independent robustness analyses: continuous length‐effect analysis, data‐adaptive quartile grouping, and exhaustive scan of valid two‐cutpoint partitions. All of them supported the same short‐sequence degradation trend (Figures S1 and S2).

### 2.2 Binding‐Supervised Retrieval Restores Target‐Aligned Peptide Geometry

**FIGURE 3:**
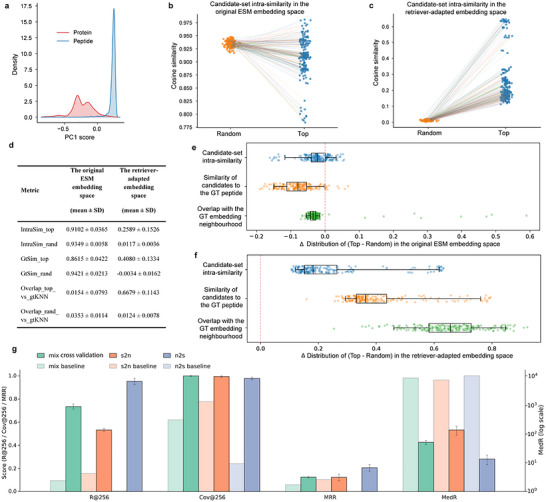
Original ESM peptide embeddings collapse in geometry, whereas binding supervision restores target‐aligned local structure for retrieval. (a) Distribution of first principal component (PC1) scores of ESM embeddings for proteins and peptides. Peptide embeddings concentrate along the dominant component, whereas protein embeddings remain broadly distributed. (b, c) Candidate‐set intra‐similarity for *K* = 256 peptides per query in the original ESM embedding space (b) and in the retriever‐adapted embedding space (c). For each query protein, Top denotes the top‐K peptides and Random a size‐matched uniformly sampled peptide set. Points represent 200 randomly sampled strict‐domain query proteins used only for this locality analysis; lines connect paired comparisons for the same query. (d) Summary of locality metrics in the original ESM and retriever‐adapted spaces. IntraSim denotes the mean pairwise cosine similarity within each set. GtSim denotes the mean cosine similarity between each candidate peptide and its ground‐truth. Overlap_vs_gtKNN denotes the overlap between the candidate set (Top or Random) and the ground truth neighborhood. (e, f) Per‐query differences (Top − Random) for locality metrics in the original ESM space (e) and the retriever‐adapted space (f). Boxplots show the median and interquartile range. Red dashed lines indicate zero difference. (g) Performance of our target‐conditioned retriever and an ESM baseline across three evaluation protocols: n2s (train on noisy, test on strict), s2n (train on strict, test on noisy) and mix (noisy + strict with cross‐validation). Recall@256, Coverage@256 and mean reciprocal rank (MRR) are plotted on the left y axis (range 0–1 and higher is better), whereas median rank (MedR) is plotted on the right axis (log scale and lower is better). Bars show mean ± s.d. across 10 repeats.

We found that ESM‐2 peptide embeddings undergo a marked collapse, rendering distinct peptides nearly indistinguishable in representation space. In the global ESM embedding space, peptide representations concentrate tightly along the dominant principal component, whereas protein representations remain broadly distributed (Figure 3a). This compressed geometry implies that local operations in ESM space of peptides, such as nearest‐neighbor search or embedding‐space perturbation, will be poorly aligned with meaningful biological variation and instead behave as quasi‐random jitter within that collapsed region of the space.

We therefore examined nearest‐neighbor search as the most direct test of locality in the ESM peptide embedding space. This locality diagnostic used 200 randomly sampled strict‐domain query proteins, each paired with a ground‐truth (GT) peptide. For each query, we then retrieved the top‐256 peptides from the library as its candidate set (Top) by ranking peptides by cosine similarity to the protein embedding. As a control, we also sampled an equally sized peptide set uniformly at random (Random). We found that candidate‐set intra‐similarity was extremely high for both Top and Random sets (0.9102 ± 0.0365 vs. 0.9349 ± 0.0058, Figures 3b,e), consistent with peptides occupying a narrowly concentrated region of the ESM embedding space irrespective of selection strategy. More strikingly, the Top retrieval did not enrich proximity to the GT peptide. The mean cosine similarity to GT was even lower for the Top set than for the Random set (0.8615 ± 0.0422 vs. 0.9421 ± 0.0213). Likewise, the overlaps between the candidate sets and the GT KNN neighborhood (the 256 nearest peptide neighbors of the GT peptide in ESM space) were both near zero (Figure 3e and Figures S3,S4). The lack of enrichment was also reflected at the sequence level for both Jaccard similarity and BLOSUM62‐based similarity (Tables S1–S3). These results indicate that distances in the raw peptide embedding space are not operationally meaningful under such collapse.

Despite the collapse of raw peptide geometry in ESM space, binding‐pair supervision can still yield effective discriminators for protein–peptide matching, as demonstrated by CLIP‐style dual‐encoder models [2]. Yet, it remains unclear how such supervision reshapes peptide representations and whether the resulting geometry supports scalable library indexing and fast querying. We therefore developed a target‐conditioned peptide retriever (Figure 1) to interrogate what binding supervision is learning.

Across evaluation protocols, our retriever showed robust and practically useful performance, consistently outperforming the ESM baseline (Figure 3g). We evaluated three settings: n2s (trained on the noisy dataset and tested on strict); s2n (the reverse direction) and mix (merging the noisy and strict datasets followed by cross‐validation on protein clusters), and each setting was repeated 10 times. Performance was assessed using standard information‐retrieval metrics. Because the noisy and strict datasets are constructed using different filtering criteria, these protocols impose different degrees of distribution shift and difficulty: n2s is comparatively easier, whereas s2n is the most challenging. Nevertheless, our retriever maintained strong performance across all settings, supporting that it captures generalized structure across distributions. We further examined the sensitivity of performance to the choice of retrieval depth K (Figure S5). Balancing retrieval quality against downstream computational cost, we set K to 256.

We then asked what binding supervision changes in representation space. To test whether performance reflects improved local geometry rather than scoring alone, we repeated our locality analysis described above in the retriever‐adapted embedding space. The results revealed a pronounced geometric “de‐collapse.” In the retriever‐adapted embedding space, Random sets became effectively unstructured, while Top sets formed coherent, query‐specific neighborhoods, with intra‐set similarity as 0.0117 ± 0.0036 vs. 0.2589 ± 0.1526 (Figure 3c,f). Crucially, this coherence aligned with ground truth. Top candidates showed substantially higher similarity to the GT peptide than Random (0.4080 ± 0.1334 vs. −0.0034 ± 0.0162, paired Wilcoxon p = 1.44 × 10^−^^3^ ^4^, Figure 3f and Figure S3) and recovered the GT local neighborhood with significantly higher overlap (0.6679 ± 0.1143 vs. 0.0124 ± 0.0078, p = 1.43 × 10^−^^3^ ^4^, Figure 3f and Figure S4). This improvement was also reflected at the sequence level, arguing against a purely geometric artefact. Retrieved Top peptides exhibited significantly higher internal sequence coherence than Random and substantially greater sequence similarity to the GT peptide (Tables S1–S3). These results show that our retriever expands the effective volume of peptide representations and restores discriminative, target‐aligned local structure, enabling scalable offline indexing and fast online querying.

### 2.3 Topology‐Conditioned Alignment Converts Retrieved Evidence Into Controllable Decoding States

**FIGURE 4:**
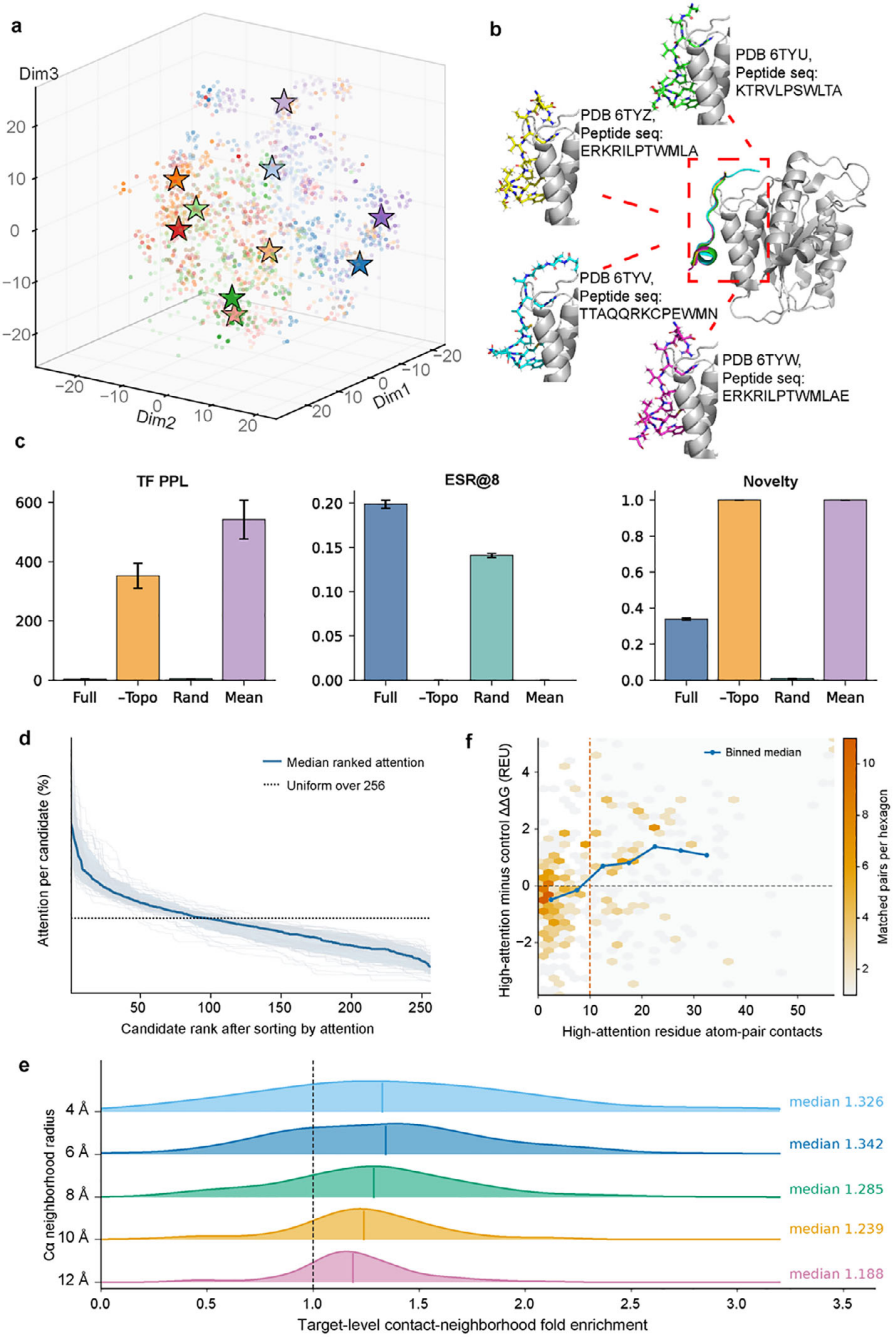
Topology‐conditioned bipartite alignment yields residue‐level preference states for evidence‐grounded peptide decoding. (a) 3D t‐SNE visualization of the frozen retriever embedding space for proteins (stars) and peptides (dots). For each protein target, associated peptides (same color) occupy a local neighborhood around the target representation. (b) A single target can bind multiple distinct peptides, illustrated by four complexes with the same receptor fold and different bound peptides. Receptor is colored by grey cartoon; peptides are shown as colored sticks. (c) Ablation of topology conditioning. Teacher‐forcing perplexity (TF PPL), exact sequence recovery among the top‐8 generated peptides (ESR@8) and novelty are shown for the full model (“Full”), a model without topology conditioner (“–Topo”), a model using random context peptides (“Rand”) and a model using mean‐pooling in place of the topology conditioner (“Mean”). All comparisons use identical training and decoding settings. Bars indicate variability across independent runs. (d) Ranked candidate‐level attention profiles across held‐out targets. Attention weights over the *K* = 256 retrieved peptide candidates were normalized to sum to one and sorted in descending order. Grey curves show individual targets and the blue curve shows the median ranked profile. The dotted line indicates a uniform distribution over 256 candidates. (e) Multiscale enrichment of high‐attention protein residues near peptide‐contact neighborhoods. Ridgeline densities show the target‐level fold enrichment of top‐10% protein‐side attention residues within 4, 6, 8, 10 and 12 Å Cα neighborhoods of direct peptide‐contact residues. (f) Relationship between interface contact richness and paired alanine‐scan effect. Each matched pair is positioned by the high‐attention residue's atom‐pair contact count and its high‐attention‐minus‐control Rosetta ΔΔG. Hexagon color indicates pair density and the blue line shows binned medians. The vertical dashed line marks the predefined ≥10‐contact subset and the horizontal dashed line mark no energetic difference.

We first examined the organization of peptide binders in the retriever embedding space. For a given target protein, experimentally supported binders concentrate in a local neighborhood around the target representation (Figure 4a), suggesting that binding‐competent sequences occupy a target‐specific local manifold with similar sequence patterns and physicochemical profiles rather than being uniformly distributed in sequence space. Consistent with this, multiple distinct peptides can bind the same target protein, as illustrated by Figure 4b. Together, these observations motivate a generation strategy that anchors decoding to locally relevant evidence, while still allowing controlled departures from any single retrieved exemplar.

We therefore instantiate this concept as a topology‐conditioned bipartite alignment module that converts the retrieved peptides into an explicit, protein‐centric conditioning state (Figure 1). To determine whether this topology conditioning is necessary for practical peptide sequence generation, we first performed training‐level ablations that isolate the effect of the topology‐conditioned alignment while keeping the same retriever, decoding budget, and evaluation protocol. As shown in Figure 4c, replacing target‐relevant retrieved context with random peptide context (“Rand”), removing the topology conditioner (“–Topo”), or replacing topology‐aware message passing with mean pooling (“Mean”) all reduced generation performance relative to the full model (“Full”). To further test whether the trained full model depends on retrieved and topology‐conditioned evidence at inference time, we then performed fixed‐checkpoint perturbation controls. Across all three perturbations, removing peptide‐context information, mismatching the retrieved context to another target, or shuffling topology edges consistently reduced sequence recovery and increased teacher‐forced pseudo‐perplexity. Additional supporting data and complementary analyses are provided in Figures S6,S7 and Tables S4, S5. These analyses support that retrieval relevance, target‐context matching, and topology‐conditioned edge assignment are key contributors to BOND‐PEP performance.

To interpret the representations learned by the topology‐conditioned alignment module, we first examined how the model distributed attention across retrieved candidates. As shown in Figure 4d, attention over the K = 256 candidate peptides exhibits a distributed usage pattern, supporting an evidence‐grounded mechanism that integrates signals across multiple candidates rather than memorizing one exemplar. We then tested whether the attention‐derived protein‐side preference score was spatially associated with peptide‐contact regions across targets. For the top‐10% attention residues, the median fold enrichment over target‐specific background remained consistently above 1 for 163/193 targets across 4, 6, 8, 10, and 12 Å Cα‐neighborhood definitions (Figure 4e). Additional distance and attention‐decile diagnostics supported the same spatial association (Figure S8). A representative case with structural mapping is provided in Figure S9.

We next tested whether contact‐rich protein‐side interface residues with high attention values exhibited hotspot‐like energetic and interaction signatures. We performed Rosetta alanine scanning on high‐attention direct‐interface residues and matched low‐attention interface controls (Figure 4f). For high‐attention residues with at least 10 protein‐peptide atom‐pair contacts, alanine mutation produced larger predicted destabilization than in matched controls, with median ΔΔG values of 1.679 vs. 0.590 REU (Holm‐adjusted p = 6.71 × 10^−9^). The result was similar for residues with contact counts at or above the target‐level interface median (1.511 vs. 0.641 REU; Holm‐adjusted p = 3.60 × 10^−8^). A complementary non‐covalent interaction audit showed the same trend (Figure S10). These contact‐rich residues with high attention values had more heavy‐atom and side‐chain contacts, contacted more peptide residues, and were closer to the peptide than their matched controls.

Finally, we linked the protein‐side attention score to three‐dimensional peptide‐contact geometry by constructing a protein‐residue × peptide‐residue contact map for each target (Figure S11). Under the primary 4.5 Å heavy‐atom definition, the top‐10% attention residues captured a disproportionate share of contact‐map edges, with a median edge fold enrichment of 1.556 (sign‐test p = 8.05 × 10^−7^). The result remained similar at 5.0 and 6.0 Å (median enrichments, 1.562 and 1.498) and under top‐10‐residue and top‐5% attention definitions (1.500 and 1.714, respectively).

### 2.4 BOND‐PEP Improves Sequence‐Only Peptide Binder Generation on Unseen Targets

**FIGURE 5:**
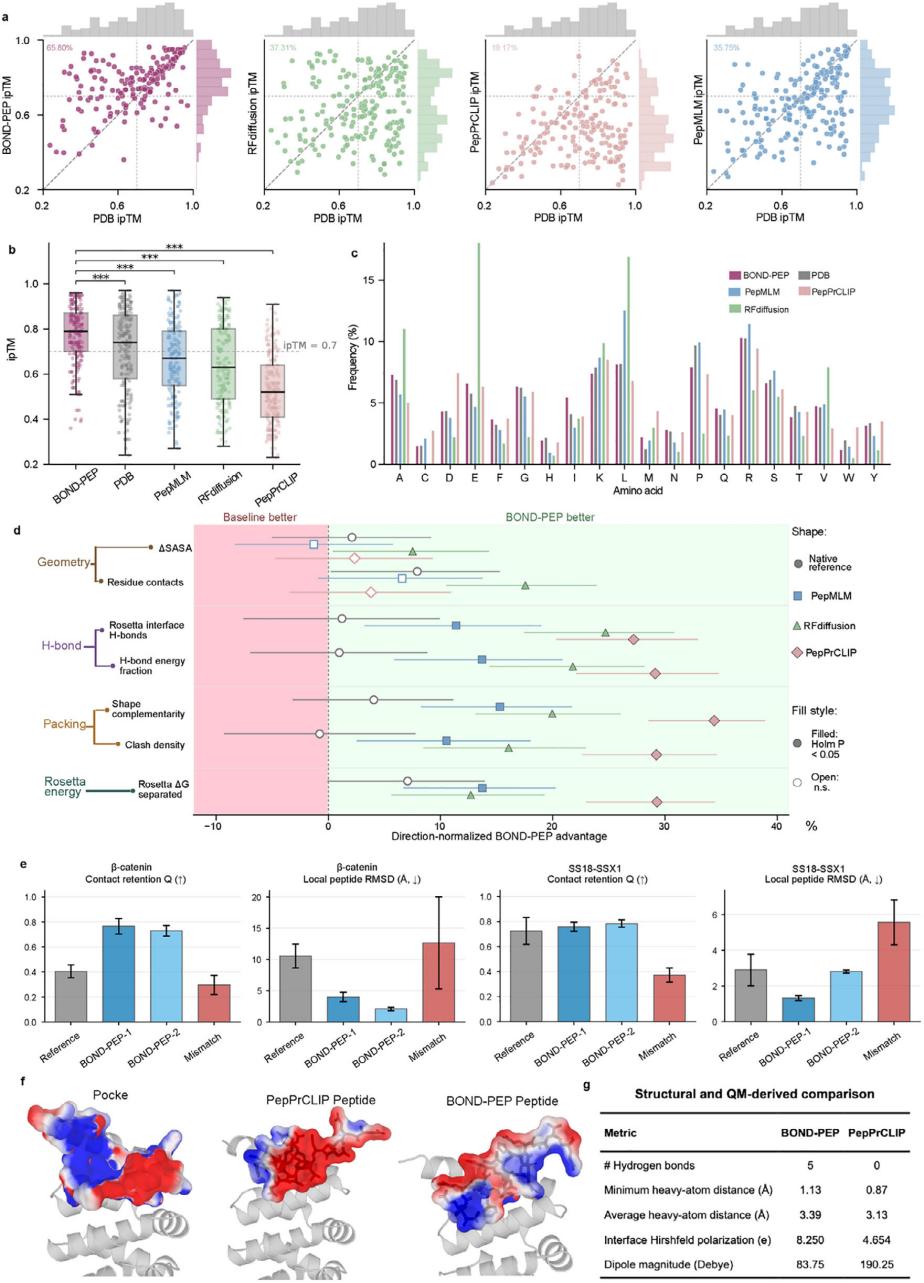
Benchmarking and representative interface analyses of BOND‐PEP. (a) In silico reference‐beating ipTM success assessment of BOND‐PEP (leftmost), RFdiffusion (second‐from‐left), PepPrCLIP (second‐from‐right) and PepMLM (rightmost) on the unseen non‐homologous held‐out strict test set. For each target, the highest ipTM of the peptide generated by each method was compared with that of the corresponding PDB reference. Points above the diagonal indicate targets for which the designed peptide achieved a higher ipTM than the reference. Percentages indicate reference‐beating ipTM success. Marginal histograms summarize the ipTM distributions of the PDB reference peptides (top) and designed peptides (right). (b) Distribution comparison of ipTM values across the held‐out set for BOND‐PEP, PDB reference peptides, PepMLM, RFdiffusion, and PepPrCLIP. Each point represents one target. Statistical comparisons are indicated above the boxes: ^***^ *p* < 0.001. (c) Amino‐acid frequency distributions of PDB reference peptides and peptides designed by BOND‐PEP, PepPrCLIP, PepMLM and RFdiffusion on the held‐out set. (d) Forest plot showing the direction‐normalized BOND‐PEP advantage over PDB reference, PepMLM, RFdiffusion, and PepPrCLIP across representative non‐ipTM interface‐quality metrics. For each metric and comparator, the effect is defined as the fraction of targets for which BOND‐PEP performs better among all targets with non‐tied paired outcomes minus 50%, after orienting each metric so that positive values indicate better BOND‐PEP performance. Whiskers indicate Wilson 95% confidence intervals. Filled markers denote Holm‐adjusted Wilcoxon signed‐rank *p* < 0.05 while open markers denote non‐significant comparisons. Pale red and pale green backgrounds indicate comparator‐better and BOND‐PEP‐better regions, respectively. (e) Representative short‐timescale MD simulations for external targets. Bars with error bars show the final‐window summary across three independent 30 ns simulations per complex. Contact retention Q measures the fraction of initial protein‐peptide residue contacts retained during the trajectory and is favorable when higher. Local peptide RMSD measures peptide displacement relative to the initial binding pose after alignment on the protein interface and is favorable when lower. (f) Electrostatic surface potential of the β‐catenin binding pocket (left), the PepPrCLIP peptide (middle) and BOND‐PEP peptide (right). Shown in top view, red and blue indicate negative and positive electrostatic potentials, respectively. (g) Structural and quantum mechanics (QM)‐derived comparison between the BOND‐PEP and PepPrCLIP β‐catenin complexes.

We then benchmarked BOND‐PEP on the non‐homologous held‐out strict set, which contains 193 protein‐peptide pairs that remained fully unseen during model development. Following the same AlphaFold‐Multimer protocol across methods, we co‐folded each target–peptide pair and used the highest interface predicted Template Modeling score (ipTM) among the final five outputs as a proxy for interface quality. For each target, we compared the top peptide generated by each method against the corresponding PDB reference peptide (Figure 5a). Here, we defined reference‐beating ipTM success@8 as a target‐level success when at least one of the top‐8 generated peptides achieved an ipTM higher than the corresponding PDB reference peptide under the same co‐folding protocol. Under this stringent evaluation, BOND‐PEP achieved 65.80% reference‐beating ipTM success@8, outperforming RFdiffusion (37.31%), PepPrCLIP (19.17%), and PepMLM (35.75%). Moreover, BOND‐PEP designed peptides occupied the region above the diagonal over a broader range of target difficulties compared with other methods, indicating a global improvement rather than one driven by a few favorable cases. Beyond the structural comparison of the top peptide, we also quantified diversity within the eight BOND‐PEP candidates generated for each target. On the held‐out strict benchmark, the median per‐target unique‐sequence rate was 1.000, and the median mean pairwise sequence diversity was 0.826 (Table S6).

This advantage was also evident at the distribution level. Across the held‐out set, BOND‐PEP showed the highest overall ipTM distribution, with a large fraction of designs concentrated in the 0.7–0.9 range, whereas PepPrCLIP, PepMLM and RFdiffusion were all shifted toward lower‐confidence interfaces (Figure 5b). As ipTM > 0.7 is commonly taken as in silico evidence of effective binding, these results indicate that BOND‐PEP moved a substantially larger fraction of designs into the effective‐binding regime. Notably, in this proxy‐based comparison, the BOND‐PEP distribution was slightly shifted above that of the PDB reference peptides (Figure 5b), suggesting that its improvement extended beyond other in‐silico generators to many experimentally resolved target–peptide pairs. At the sequence‐composition level, BOND‐PEP peptides remained broadly consistent with the PDB references across most amino acids (Figure 5c). PepPrCLIP and PepMLM showed a similarly plausible overall composition, whereas RFdiffusion displayed a stronger compositional skew, suggesting BOND‐PEP improves binding‐oriented generation while remaining within the composition range of canonical amino‐acid peptide sequences represented in the benchmark.

To test whether this improvement was supported beyond ipTM, we performed a systematic interface‐quality analysis including 17 non‐ipTM metrics on the held‐out set (Table S7). These metrics covered interface burial and contact geometry, hydrogen‐bond and polar compatibility, packing and shape complementarity, and Rosetta InterfaceAnalyzer‐derived energy‐like terms. As shown in Figure 5d, relative to the PDB reference, BOND‐PEP achieved lower Rosetta separated interface energy (ΔG_separated; 464.4 vs. 522.6 REU) and slightly higher shape complementarity (0.672 vs. 0.652). Across three compared methods, BOND‐PEP consistently showed lower Rosetta ΔG_separated and higher shape complementarity, with the largest improvements over PepPrCLIP (82.1% lower ΔG_separated, Holm‐adjusted p = 1.98 × 10^−17^; 59.2% higher shape complementarity, Holm‐adjusted p = 2.00 × 10^−23^).

To further assess whether these designs of BOND‐PEP introduced developability‐related liabilities, we performed a sequence‐level screen including peptide length, molecular weight, net charge at pH 7.4, Grand Average of Hydropathy (GRAVY), residue‐composition descriptors, hydrophobic stretch length, and prespecified sequence‐liability motifs (Figure S12). BOND‐PEP designs showed a generally plausible peptide‐lead physicochemical profile, with a median length of 14 amino acids, median molecular weight of 1857.2 Da, median net charge of +1.03 at pH 7.4, and median GRAVY of ‐0.50. Consistently, hydrophobicity risk, high absolute charge rate, and multiple‐cysteine risk of BOND‐PEP‐generated peptides were comparable to or lower than those of the matched PDB reference peptides. The overall fraction of peptides carrying at least one developability‐related red flag was also slightly lower for BOND‐PEP than for PDB references.

To test whether this advantage extends beyond the PDB‐derived benchmark, we next evaluated BOND‐PEP on a compact external panel of targets with previously reported experimentally supported peptide binders [2, 3]. Rather than aiming for exhaustive target coverage, we selected a compact, non‐redundant set that spans distinct functional scenarios, including direct binding, enzymatic inhibition, and degradation of structured and disordered disease targets. This set comprised β‐catenin, UltraID, SS18‐SSX1 and NCAM1, enabling direct comparison between BOND‐PEP and experimentally validated reference peptides under the same AlphaFold‐Multimer protocol. In all four cases, the peptide designed by BOND‐PEP achieved a higher ipTM than the reference (with gains ranging from 0.138 to 0.385 across targets, Table S8). For example, for β‐catenin, the ipTM increased from 0.486 for the reference peptide to 0.871 for the BOND‐PEP design. We also examined whether the external‐panel designs reflected recurrent, target‐independent peptides. BOND‐PEP showed similarly low within‐target and cross‐target similarities (0.151 and 0.164, respectively), although a near‐recurrent β‐catenin / NCAM1 candidate was present in Table S8. These similarities were comparable to the low but non‐zero background observed for other methods, consistent with the many‐to‐many nature of protein‐specific peptide design.

We further performed short‐timescale molecular dynamics (MD) simulations for representative external targets. For each target, we simulated four peptide‐complex groups under the same protocol: one experimentally supported reference peptide, the top two BOND‐PEP‐generated peptides, and one target‐peptide mismatch negative control (Figure S13). Each complex was simulated with three independent 30 ns replicas to reduce trajectory‐specific stochastic effects. In these systems, BOND‐PEP designs showed higher interface retention than mismatch controls, as reflected by higher contact retention and lower local peptide RMSD (Figure 5e), supporting target‐specific binding stability of BOND‐PEP‐generated peptides. We then used β‐catenin as a representative case for more detailed interface analysis and found that the improved performance of BOND‐PEP was accompanied by a more organized interface (Figure 5f). Electrostatic surface mapping showed clearer charge complementarity around the binding region for the BOND‐PEP complex, whereas the reference design displayed a less coherent interfacial pattern. The BOND‐PEP peptide also formed five hydrogen bonds, whereas the reference peptide formed none, despite comparable heavy‐atom contact distances, suggesting that the reference peptide was more crowded and less favorably accommodated at the interface (Figure 5g). Quantum‐chemical analysis supported the same trend. The BOND‐PEP interface showed stronger Hirshfeld polarization together with a lower overall dipole magnitude, consistent with a more localized interfacial electronic response. These results support the transferability of BOND‐PEP across targets spanning distinct functional contexts.

## 3 Discussion

Our findings suggest that a central limitation of sequence‐first peptide binder design lies not simply in generative capacity, but in how target‐relevant evidence is represented and exposed during decoding. BOND‐PEP addresses this limitation by converting empirical binding evidence into an explicit, residue‐resolved conditioning state. Its contribution therefore lies not only in improved generation, but in recasting controllability in sequence‐only peptide design, especially when structural information is unavailable, uncertain, or conformationally heterogeneous. Under a matched AlphaFold‐Multimer evaluation framework, BOND‐PEP outperformed competing methods on a strict non‐homologous held‐out set and showed encouraging transfer to a compact panel of targets with previously reported experimentally supported peptide binders.

Our results also argue that peptide design should not be treated as a straightforward extension of protein sequence modelling. The pronounced degradation of protein language models on short peptide‐length sequences indicates a regime mismatch, suggesting that naïve reuse of protein‐derived priors is insufficient for controllable peptide generation. Within this setting, retrieval is useful not because it retrieves near‐duplicates, but because binding supervision reorganizes peptide space into a target‐aligned local manifold. Topology‐conditioned alignment then makes retrieved evidence usable for decoding by converting it into an explicit and interpretable target‐specific conditioning signal.

BOND‐PEP provides a computational framework for evidence‐grounded peptide generation in sequence‐only settings. Although the present study is anchored to matched in silico evaluation and a compact external transfer panel, the retrieval‐augmented structure provides a clear route for systematic improvement as peptide evidence grows in scale and diversity. Nevertheless, direct binding and functional assays remain important future work for validating individual molecules and quantifying affinity. Besides, the current implementation focuses on canonical amino‐acid peptides, consistent with the sequence representation used in the training and evaluation data. Extending this framework to noncanonical or chemically modified residues would require expanded residue tokenization, appropriate noncanonical training data, compatible structural evaluation pipelines, and experimental validation. Beyond this residue‐alphabet extension, the structure of BOND‐PEP also provides a natural foundation for future extensions, including multi‐objective design beyond binding, and phenotype‐ or cell‐state‐aware peptide design in which transcriptomic signatures or other systems‐level readouts provide additional conditioning signals. It may also support closed‐loop discovery workflows in which experimental feedback iteratively refines both retrieval and generation.

## 4 Methods

### 4.1 Study Design

We developed BOND‐PEP, a retrieval‐augmented framework for sequence‐only peptide binder generation. Given a query protein, the model first retrieves target‐relevant peptide candidates from a large peptide library using a frozen dual‐encoder retriever. These retrieved peptides are then coupled to the query protein through a local bipartite graph, enabling message passing that converts retrieved evidence into a protein‐centric conditioning state. This topology‐conditioned representation is subsequently injected into an autoregressive peptide decoder to guide sequence generation. The overall design was motivated by two observations from our benchmark setting: protein language model priors transfer imperfectly to short peptides, and peptide binders for a given target tend to occupy target‐aligned local neighborhoods rather than being uniformly distributed across sequence space.

### 4.2 Datasets

To ensure a fair comparison under realistic distribution shift, we reconstructed the peptide–protein benchmark by following the dataset construction protocol described in previous work [2]. More specifically, peptide–protein interaction pairs were mined from experimentally resolved structures in the RCSB Protein Data Bank and filtered into two complementary datasets: a broader noisy set containing both strong and weak interactions (filtering standard: buried surface area ≥ 50 Å^2^, peptide length < 50 amino acids and target length ≥ 50 amino acids), and a smaller strict set containing only strong interactions (filtering standard: buried surface area ≥ 400 Å^2^, peptide length ≤ 25 amino acids and target length ≥ 30 amino acids). After this, we clustered target protein sequences with MMseqs2 at 30% minimum sequence identity before any data partitioning, so that homologous proteins were assigned to the same cluster and homology leakage was minimized. We reserved one split of the strict dataset (193 pairs) as a non‐homologous held‐out test set, kept it unseen during model development, and only used it exclusively for final benchmarking. All remaining data were used as clusters for cross‐validation, hyperparameter tuning, model training, and selection. To further evaluate practical transfer beyond the PDB‐derived benchmark, we assembled a small auxiliary external benchmark from peptide sequences that were experimentally assayed in recent studies [2, 3] and used it exclusively for post hoc evaluation.

### 4.3 Protein Language Model Characterization of the Peptide‐Length Regimes

To assess whether protein‐derived sequence priors transfer uniformly to the limited context of peptide‐length sequences, we evaluated ESM‐2‐650 M and ESM‐C‐600 M on proteins, peptides, and length‐matched protein fragments. For each sequence category, 1000 sequences were randomly sampled from our sequence dataset. The peptide and length‐matched protein fragment sets were grouped into ≤ 10, 11–15, and > 15 amino‐acid bins for visualization and summary. We considered three complementary tasks: self‐copy, in which the full sequence is provided and residue recovery is measured; leave‐one‐out masked recovery, which probes prediction of a held‐out residue from surrounding context; and iterative denoising from partially corrupted sequences. To test whether the observed length‐associated trends depended on this presentation scheme, we further performed continuous length‐effect analysis, data‐adaptive quartile regrouping, exhaustive two‐cutpoint scanning across valid peptide‐length partitions, and length‐matched protein‐fragment analysis using the same PLM scoring pipeline. Metric definitions and implementation details are provided in Supporting Notes.

### 4.4 Sequence Representations and Target‐Conditioned Retrieval

All proteins and peptides were represented using precomputed token‐level embeddings from ESM‐2‐650 M. During training and inference, only the embeddings required for the current minibatch were transferred from CPU to GPU. Proteins and peptides were encoded using modality‐specific sequence encoders (an attention‐based encoder for proteins and a convolutional encoder for peptides). The resulting representations were projected into a shared retrieval space using residual projection heads with multi‐subspace normalization, which stabilized similarity computation and enabled the model to reweight informative subspaces during retrieval. The retriever was trained independently from the generator using a symmetric contrastive objective over matched protein–peptide pairs, supplemented with hard‐negative mining after early training epochs. After training, the retriever was frozen, and peptide retrieval embeddings were precomputed offline for the full peptide library, enabling efficient top‐K search for any query protein. Unless otherwise stated, we set K  = 256 to balance retrieval quality against downstream computational cost. Further architectural and optimization details are provided in Supporting Notes.

### 4.5 Topology‐Conditioned Peptide Generation

Retrieved peptides were not used as independent prompts. Instead, for each query protein, we constructed a local protein‐centerd bipartite graph connecting the protein node to its top‐K retrieved peptide candidates. This topology enforced a protein‐centric evidence integration scheme in which all retrieved context was mediated through the query protein. We then applied stacked multi‐head bipartite graph‐attention layers with bidirectional updates between protein and peptide nodes. These updates distilled which retrieved candidates were informative for the current target and which protein positions were repeatedly associated with compatible peptide patterns across retrieved contexts. The resulting topology‐conditioned protein representation was injected into an autoregressive Transformer decoder as conditioning memory, thereby guiding generation through a residue‐resolved preference. Full layer definitions and message‐passing details are described in Supporting Notes.

### 4.6 Training and Inference

Generator training was performed on paired protein–peptide examples using teacher forcing. For each minibatch, proteins were encoded, the frozen retriever was used to obtain the top‐K peptide candidates, and the topology conditioner produced a query‐specific representation for downstream decoding. The primary training objective was autoregressive cross‐entropy over peptide tokens. In the reported experiments, these peptide tokens corresponded to the 20 canonical amino‐acid residues, with special tokens used only for sequence delimiting, padding, masking, and generation termination. To improve global consistency, we additionally used an auxiliary span‐masked objective during training. Model development, checkpoint selection, and ablation studies were performed using five‐fold cross‐validation. At inference time, because a single protein target may admit multiple valid peptide binders, we used multi‐candidate stochastic decoding. For each conditioned target, the model generated multiple peptide candidates by sampling from temperature‐scaled next‐token distributions after truncation of low‐probability residues. Decoding settings and auxiliary training details are given in Supporting Notes.

### 4.7 Sequence‐level Developability Analysis

We calculated peptide length and molecular weight to characterize molecular size, which is directly relevant to synthesis, formulation, delivery, and downstream optimization. We then calculated net charge at pH 7.4 because excessive positive or negative charge can affect solubility, nonspecific electrostatic interactions, membrane interaction, and assay behavior. We also calculated the Grand Average of Hydropathy (GRAVY), hydrophobic residue fraction, and the longest hydrophobic run since excessive hydrophobicity or long hydrophobic stretches may increase aggregation, poor solubility, and nonspecific binding as well. Finally, we quantified aromatic, charged, polar, and basic residue fractions to describe the overall residue composition of the generated peptides and then screened common liability motifs that may indicate chemical instability or protease sensitivity. These motifs include Cys‐rich sequences, Met/Trp‐containing oxidation‐prone motifs, Asn/Gln‐containing deamidation‐prone motifs, Asn‐X‐Ser/Thr N‐glycosylation‐like motifs, Asp‐Gly (DG) isomerization‐prone motifs, and Lys/Arg‐rich protease‐sensitive patterns.

### 4.8 Interface Analysis

We benchmarked peptides designed by different methods, together with peptides from PDB co‐crystal structures, via co‐folding each target–peptide pair with AlphaFold‐Multimer (through ColabFold using the default settings [40]). The highest ipTM score from the final 5 co‐folding outputs was used as a structure‐based proxy for interface quality. Higher ipTM values generally indicate more confident binding predictions, and according to prior work, ipTM > 0.7 could be treated as evidence of effective binding [41]. For the benchmark‐wide ipTM‐independent interface‐quality analysis, we used the predicted or reference complex structures with matched chain definitions and computed 17 non‐ipTM metrics describing interface burial and contact geometry, hydrogen‐bond and polar compatibility, packing and shape complementarity, and Rosetta InterfaceAnalyzer‐derived energy‐like terms. Geometry‐based metrics included buried SASA, ΔSASA, atom and residue contact counts, contact density and peptide‐contact fraction. Polar and packing metrics included interface hydrogen bonds, unsatisfied H‐bonds or buried polar atoms, steric clashes, shape complementarity and packstat where available. Rosetta InterfaceAnalyzer‐derived terms included ΔG_separated and related interface energy‐like components. Target‐level method comparisons were performed as paired analyses across targets using Wilcoxon signed‐rank tests, with Holm correction for multiple testing.

Short‐timescale MD stability checks were performed for representative external targets using matched simulation settings. For each target, we prepared four complex groups: the experimentally supported reference peptide, the top 2 BOND‐PEP‐generated peptides, and a target‐peptide mismatch negative control. Each complex was simulated with three independent 30 ns replicas. Stability was quantified using median‐of‐replica last‐5 ns summaries of contact retention, local peptide RMSD, protein‐peptide center‐of‐mass shift, buried SASA ratio, interchain hydrogen bonds and cross‐residue contacts. To further characterize the molecular basis of predicted peptide–protein recognition, we performed complementary electrostatic, hydrogen‐bond and quantum‐chemical analyses on representative complexes. Electrostatic surface potentials were computed using PROPKA3 [42] for protonation‐state assignment and PDB2PQR [43] for charge‐radius preparation, followed by APBS [44] for electrostatic calculations. Then, they were mapped onto molecular surfaces to qualitatively visualize charge complementarity at the peptide‐binding interface. Hydrogen bonds were identified using geometry‐based criteria [45]. For representative interfaces, we additionally carried out quantum‐chemical calculations in ORCA [46, 47] using an implicit‐water solvent model [48]. Electron‐density and electrostatic‐potential analyses from these calculations were used to assess local polarization and the electronic features of interfacial contacts [49].

## Author Contributions

W.D. conceived the study, developed the method, performed the analyses, interpreted the results, and wrote the manuscript.

## Conflicts of Interest

The author declares no conflicts of interest.

## Supporting information




**Supporting File**: advs77125‐sup‐0001‐SuppMat.pdf.

## Data Availability

The data that support the findings of this study are openly available in “*Evidence‐grounded peptide binder generation through target‐conditioned retrieval and topology‐conditioned decoding*” at https://doi.org/10.5281/zenodo.19841318, reference number Version v1.
