# Supplementary material for: Computationally Evidence‐Grounded Sequence‐First Design of Peptide Binders
Source: Adv Sci (Weinh). 2026 Aug 14:e77125. Online ahead of print. doi: 10.1002/advs.77125 (PMC13474178; doi:10.1002/advs.77125)
Supplement: Supplementary file 1 [file ADVS-9999-e77125-s001.pdf]

Evidence-Grounded Sequence-First Design of Peptide Binders

Wenze Ding

Supplementary Figures

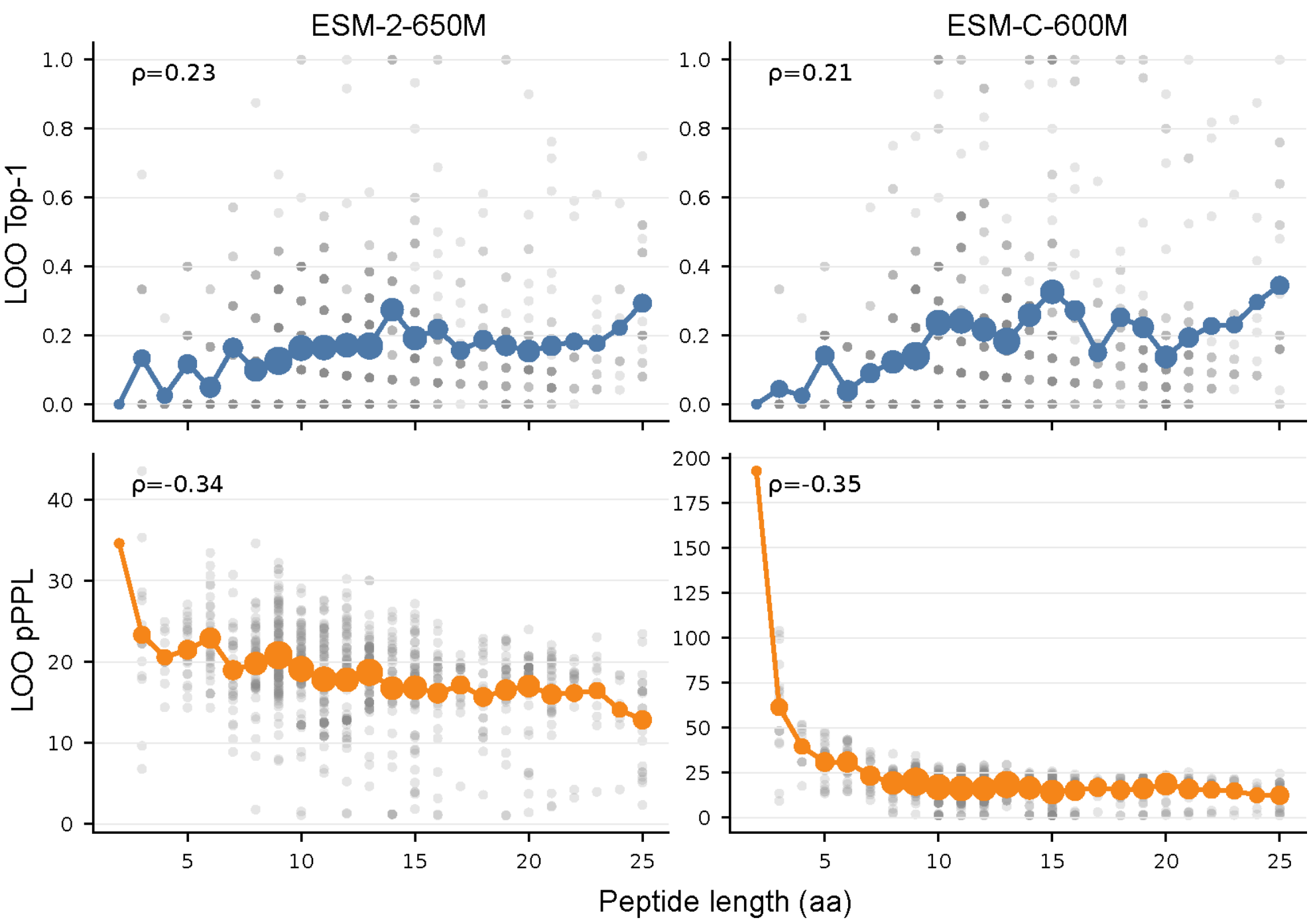

Figure S1. Continuous association between peptide length and PLM leave-one-out performance. Each grey point represents one peptide in the 1000-peptide evaluation set. Colored points show the mean value at each exact peptide length, with point size proportional to the number of peptides of that length. Top panels show leave-one-out (LOO) Top-1 recovery for ESM-2-650M (left panels) and ESM-C-600M (right panels), and bottom panels show LOO pseudo-perplexity (pPPL).

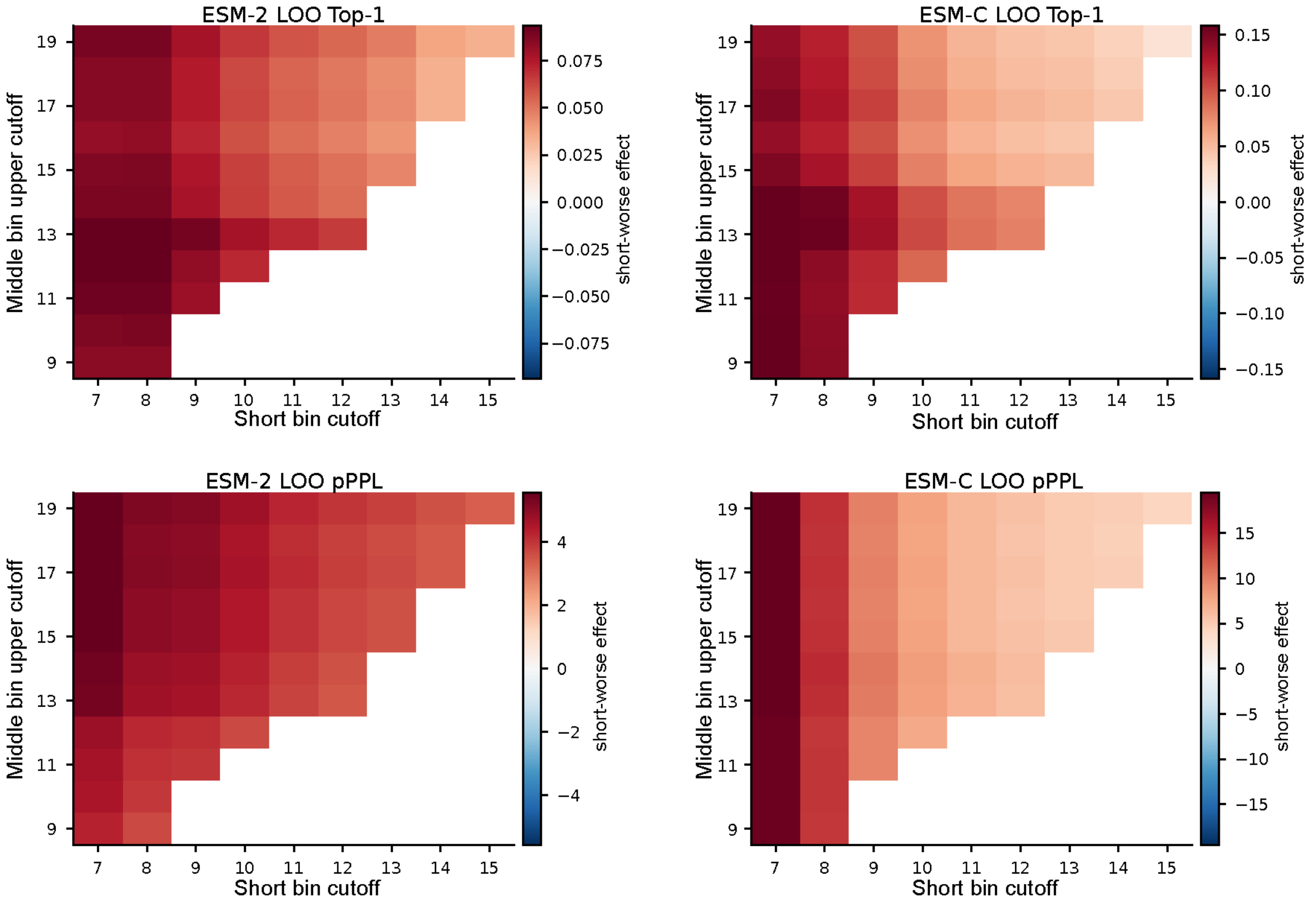

Figure S2. Exhaustive scan of two-cutpoint peptide-length partitions. Heatmaps summarize all valid two-cutpoint partitions of the 1000-peptide evaluation set, requiring at least 100 peptides in each resulting bin. The x axis indicates the short-bin cutoff and the y axis indicates the middle-bin upper cutoff. Color denotes the short-worse effect, defined so that positive values indicate lower LOO Top-1 recovery or higher LOO pPPL in shorter peptides compared with longer peptides. Across all 62 valid partitions, the short-worse direction was preserved in both ESM-2-650M and ESM-C-600M.

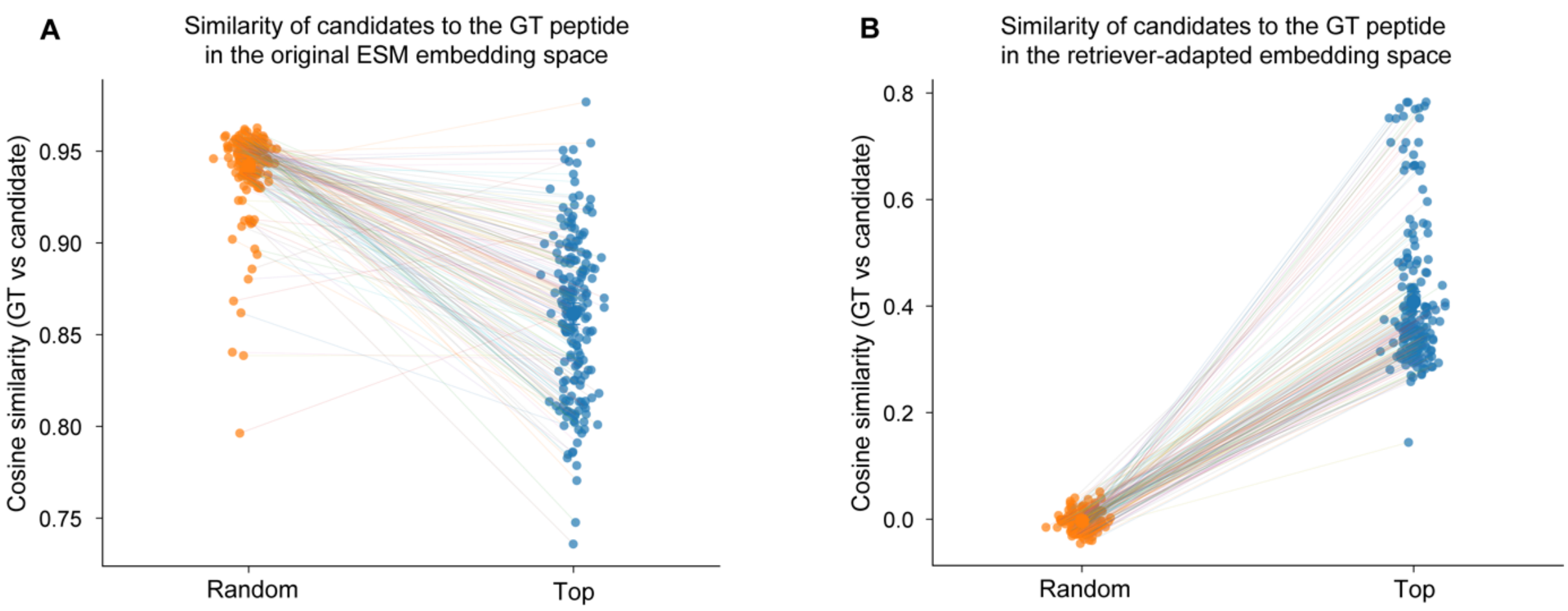

Figure S3. Similarity of candidate peptides to the ground-truth (GT) peptide in the original ESM (A) and retriever-adapted embedding spaces (B). Each dot represents one query protein (n=200). Lines connect paired Random and Top values for the same query. The 200 query targets used here are the same locality-analysis subset used in Fig. 3 and are distinct from the 193-pair final benchmark and the external panel.

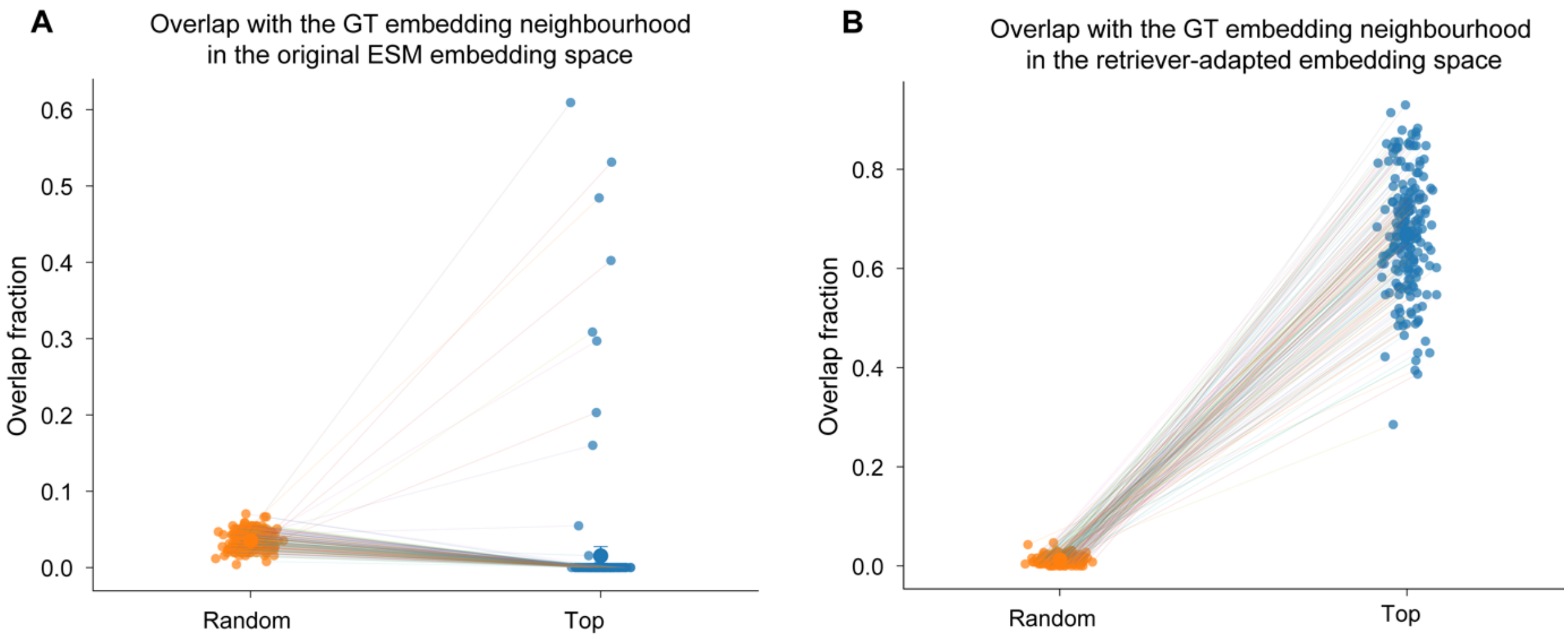

Figure S4. Overlap between retrieved candidates and the ground-truth (GT) peptide neighbourhood in the original ESM (A) and retriever-adapted embedding spaces (B). Each dot represents one query protein (n=200). Lines connect paired Random and Top values for the same query. The 200 query targets used here are the same locality-analysis subset used in Fig. 3 and are distinct from the 193-pair final benchmark and the external panel.

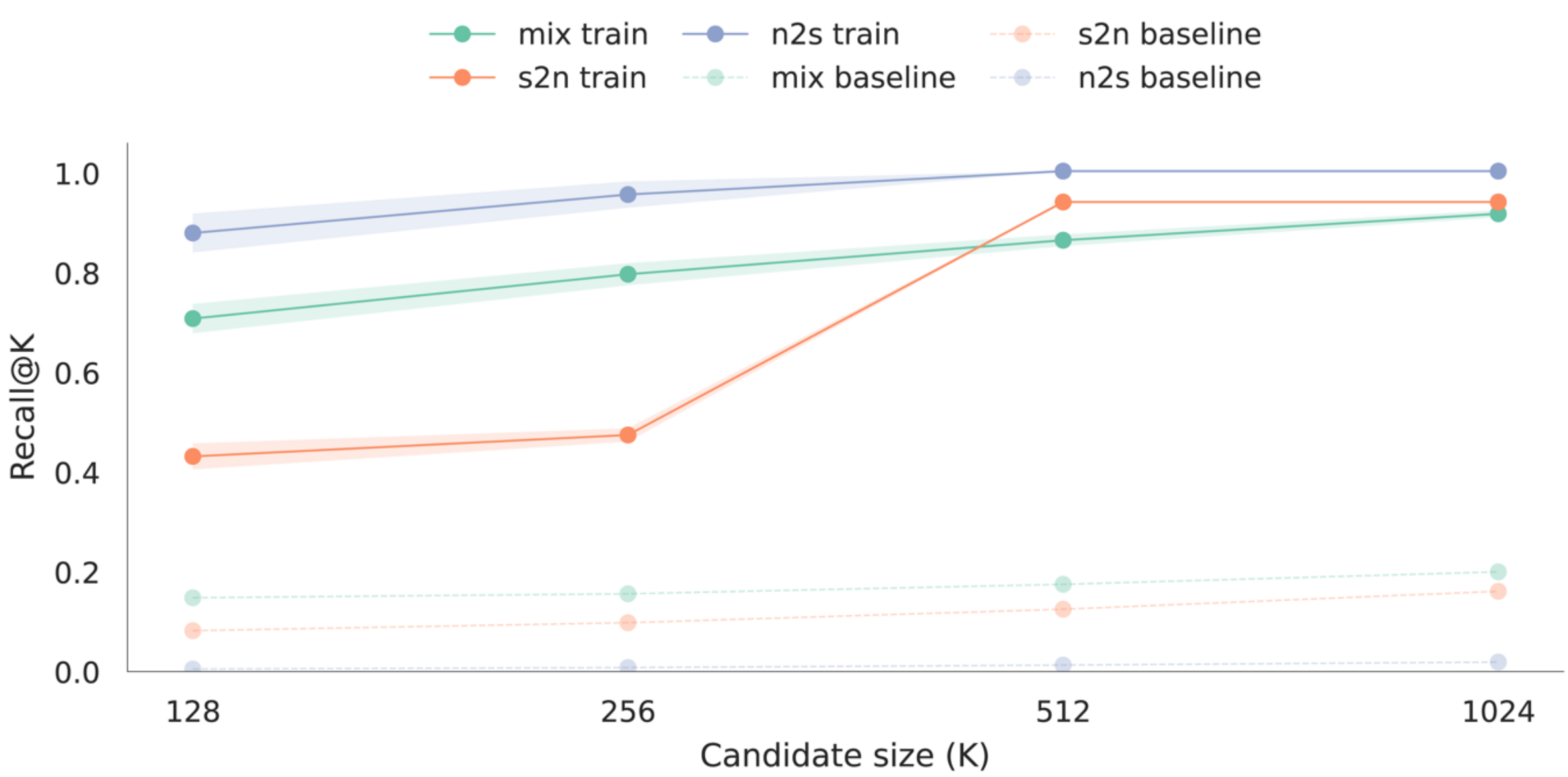

Figure S5. Retrieval performance as a function of candidate-set size. Recall@K is plotted against retrieval depth (K = 128, 256, 512 and 1,024) for our retriever (solid lines) and the ESM baseline (dashed lines) under three evaluation protocols: n2s (train on noisy, test on strict), s2n (train on strict, test on noisy) and mix (noisy + strict with cross-validation). Points indicate the mean across 10 repeats and shaded regions denote s.d.

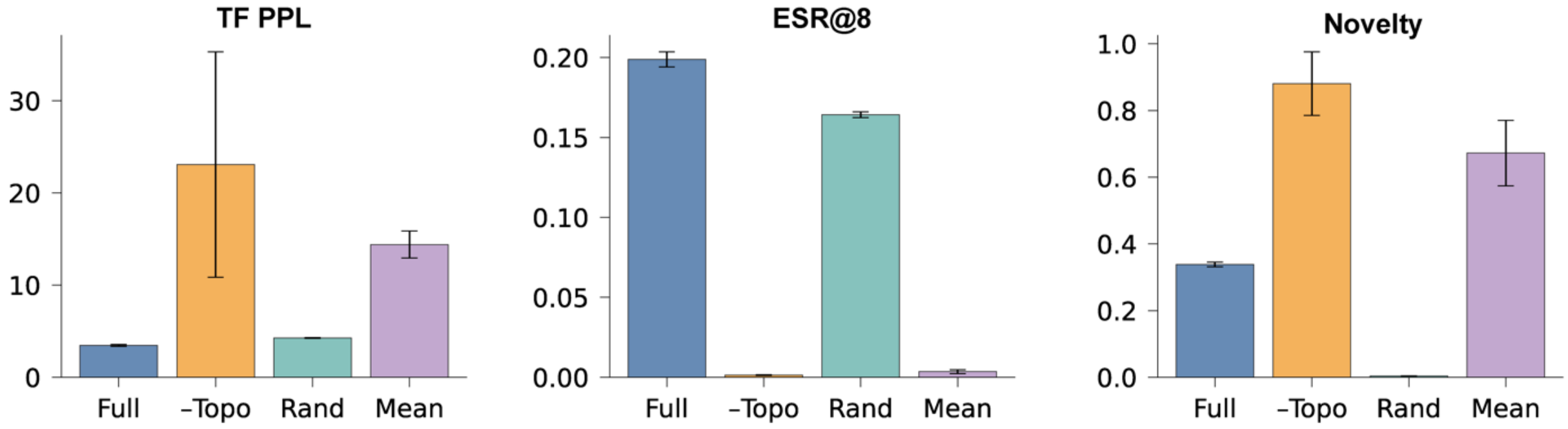

Figure S6. Ablation of topology conditioning using best epochs. Teacher-forcing pseudo-perplexity (TF PPL), exact sequence recovery among the top-8 generated peptides (ESR@8) and novelty for the full model ("Full") versus removing the topology conditioner ("–Topo"), using a randomly selected context instead of the retrieved one ("Rand") or using mean-pooling in place of the topology conditioner ("Mean"). All comparisons use identical training and decoding settings, and metrics are reported using the best training epoch for each variant. Error bars indicate variability across independent runs.

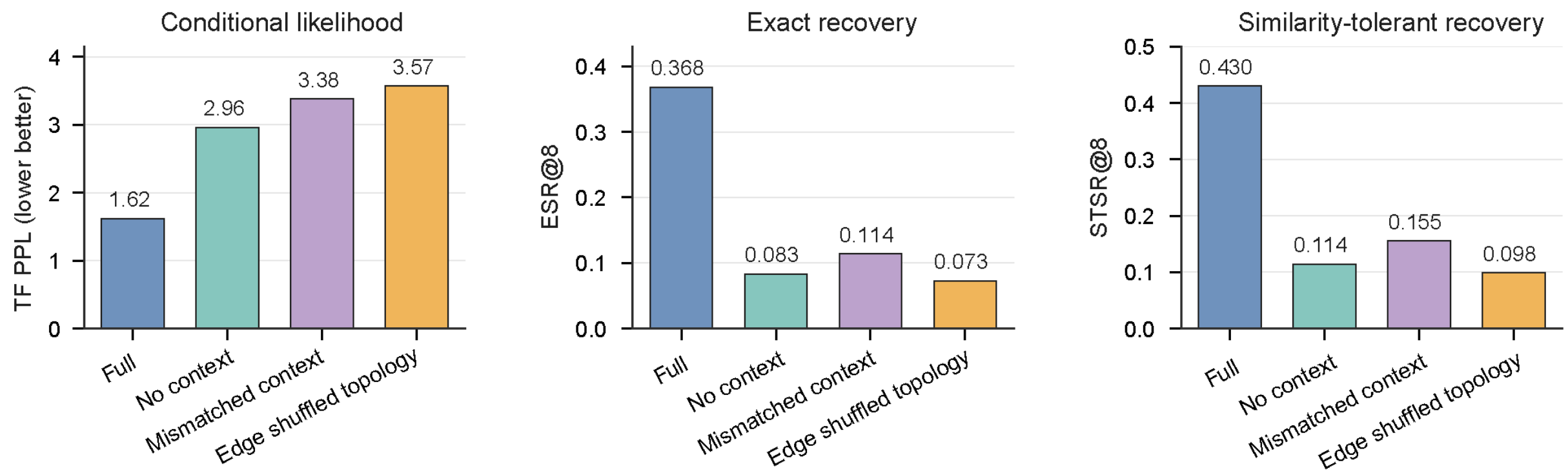

Figure S7. Fixed-checkpoint perturbation of the full BOND-PEP model. The trained full BOND-PEP checkpoint was kept fixed, and only the conditioning pathway was perturbed at inference time. The intact full model used target-matched retrieved peptide context and topology-conditioned message passing. In the "No context" perturbation, peptide-context embeddings were zeroed while preserving graph size. In the "Mismatched context" perturbation, the query target was kept unchanged but peptide context was retrieved from another held-out target. In the "Edge-shuffled topology" perturbation, the correct retrieved peptide nodes were preserved but protein-peptide topology edges were permuted. TF PPL denotes teacher-forced pseudo-perplexity; lower values indicate better conditional likelihood. ESR@8 denotes exact sequence recovery among the top-8 generated peptides. STSR@8 denotes similarity-tolerant sequence recovery among the top-8 generated peptides.

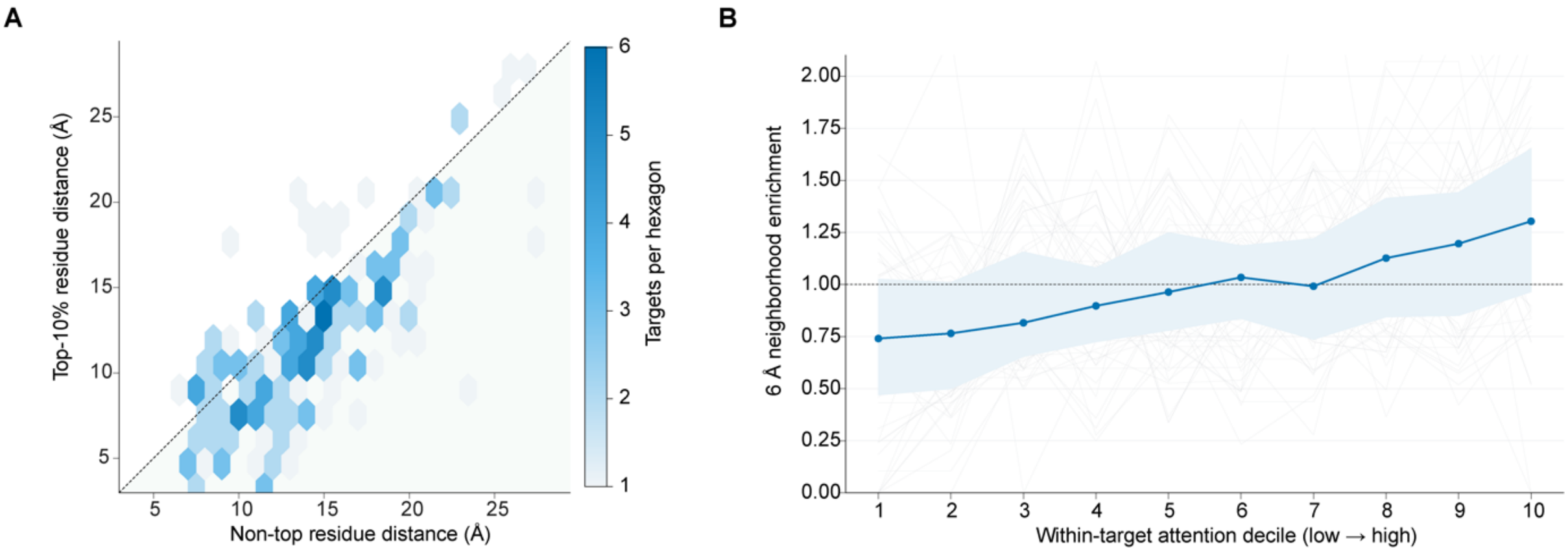

Figure S8. Protein-side attention is statistically and spatially enriched near peptide-contact regions. **(A)** Paired target-level comparison of protein-residue distance to the peptide. Each target contributes one pair of median heavy-atom distances for top-10% attention residues and non-top residues. Hexagon color indicates the number of targets. The dashed diagonal denotes equality, and points below it indicate shorter distances for top-attention residues. **(B)** Peptide-neighborhood enrichment increases with protein-side attention rank. Protein residues were divided into within-target attention deciles, and 6 Å Cα-neighborhood enrichment was calculated for each decile. The blue line shows the target-level median, the shaded region shows the interquartile range, and faint lines show a representative sample of individual target trajectories.

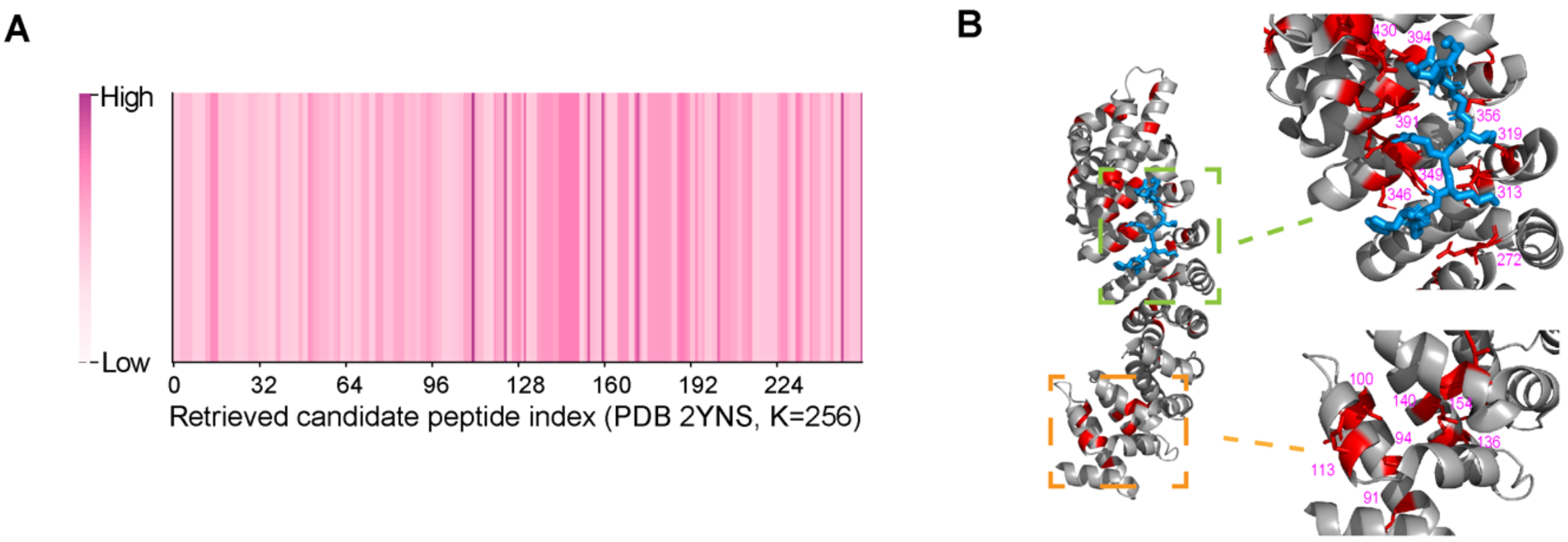

Figure S9. Representative case (PDB 2YNS) showing attention distribution of retrieved peptides and protein side high-attention residues. **(A)** Preference over retrieved peptides (K=256), indicating distributed evidence usage rather than dependence on few exemplars. **(B)** Structural mapping of high-preference residues (red) onto the receptor (grey) with the bound peptide (blue). "High-preference" residues are defined as the top-25 protein-side residues ranked by normalized attention. For plot-space constraints and visual readability, 16 residue indices were labelled in purple. The interface-proximal region (dashed green outline) and a distal region (dashed yellow outline) are highlighted and shown as zoomed insets on the right.

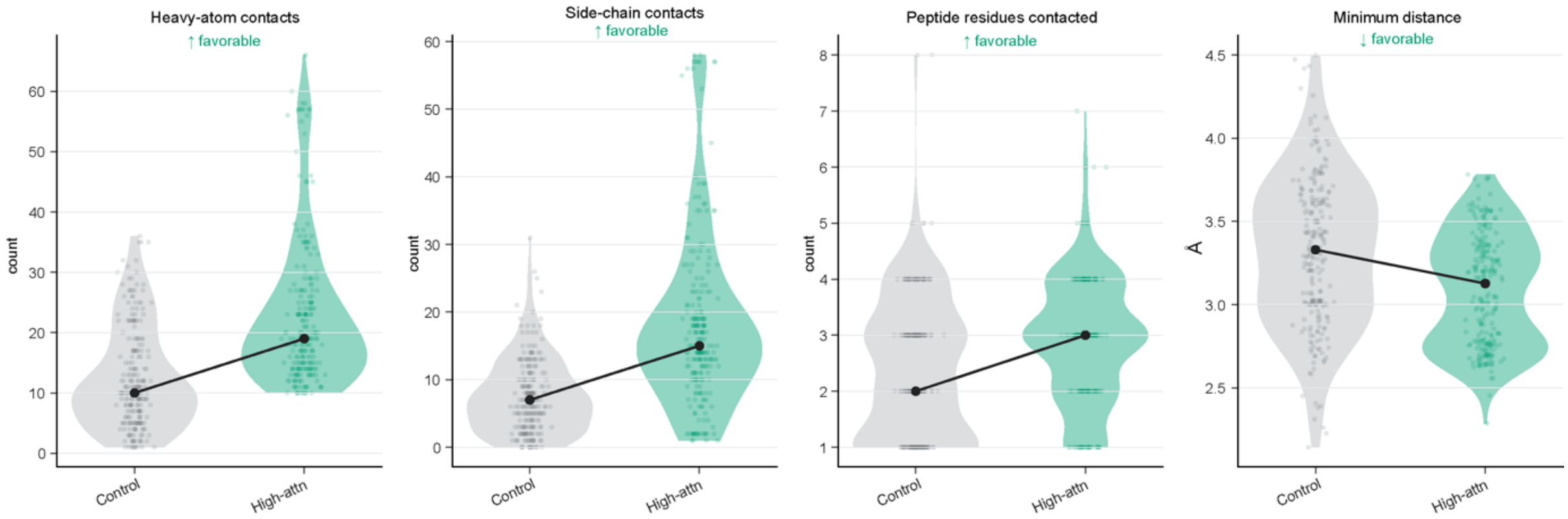

Figure S10. Non-covalent interface geometry of contact-rich high-attention residues and matched controls. Violin distributions and paired median connectors summarize heavy-atom contacts, protein side-chain contacts, contacted peptide residues and minimum heavy-atom distance in the ≥10-contact subset. Higher contact counts and lower distance indicate stronger local interface engagement.

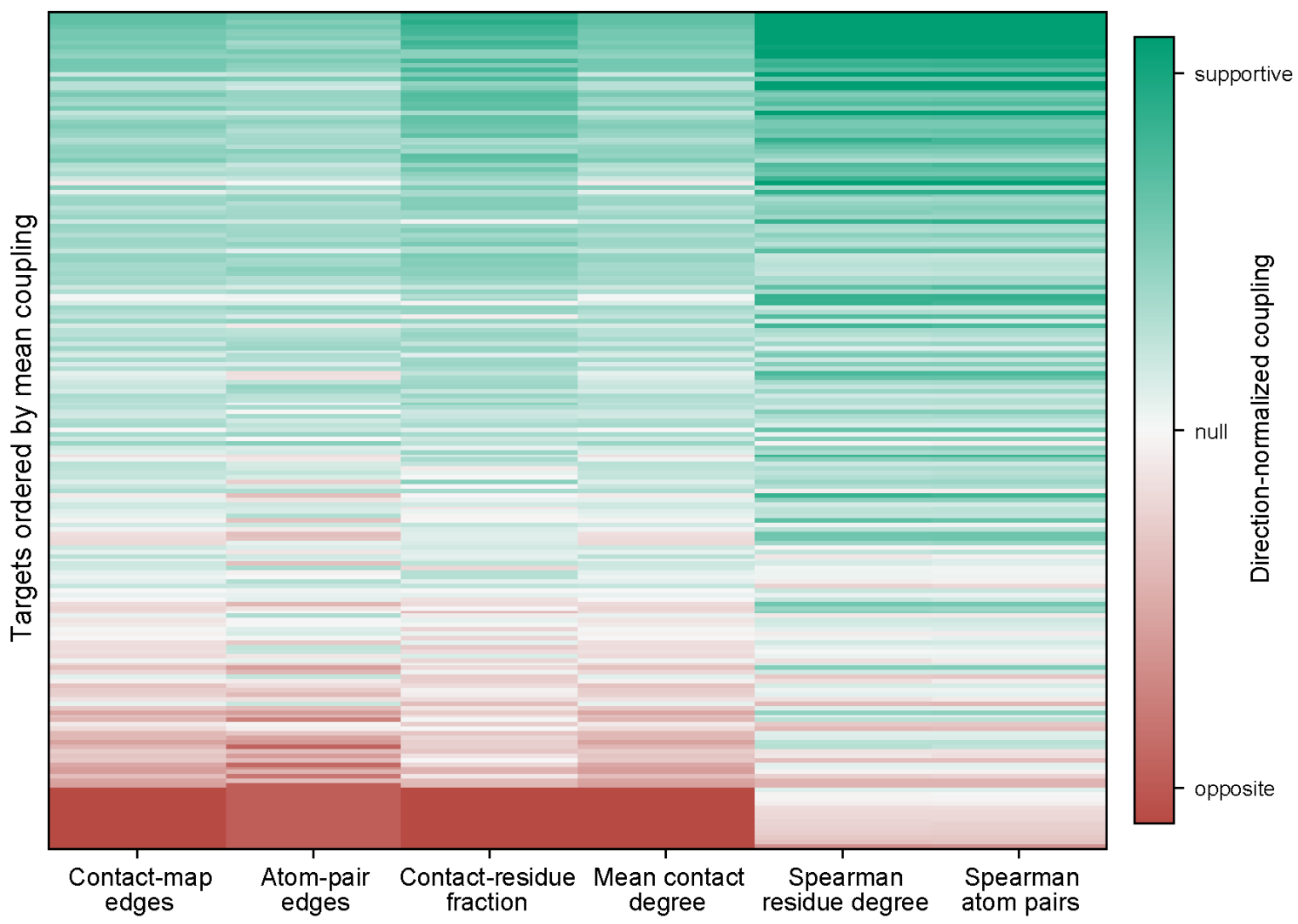

Figure S11. Target-resolved atlas of contact-map coupling. Rows represent targets ordered by mean coupling and columns show contact-map edge, atom-pair edge, contact-residue fraction, mean contact-degree and two continuous attention-degree correlation metrics under the primary 4.5 Å/top-10% definition. Values were direction-normalized within each metric; green denotes supportive coupling and red the opposite direction. Color intensity is not comparable as a physical effect size between columns.

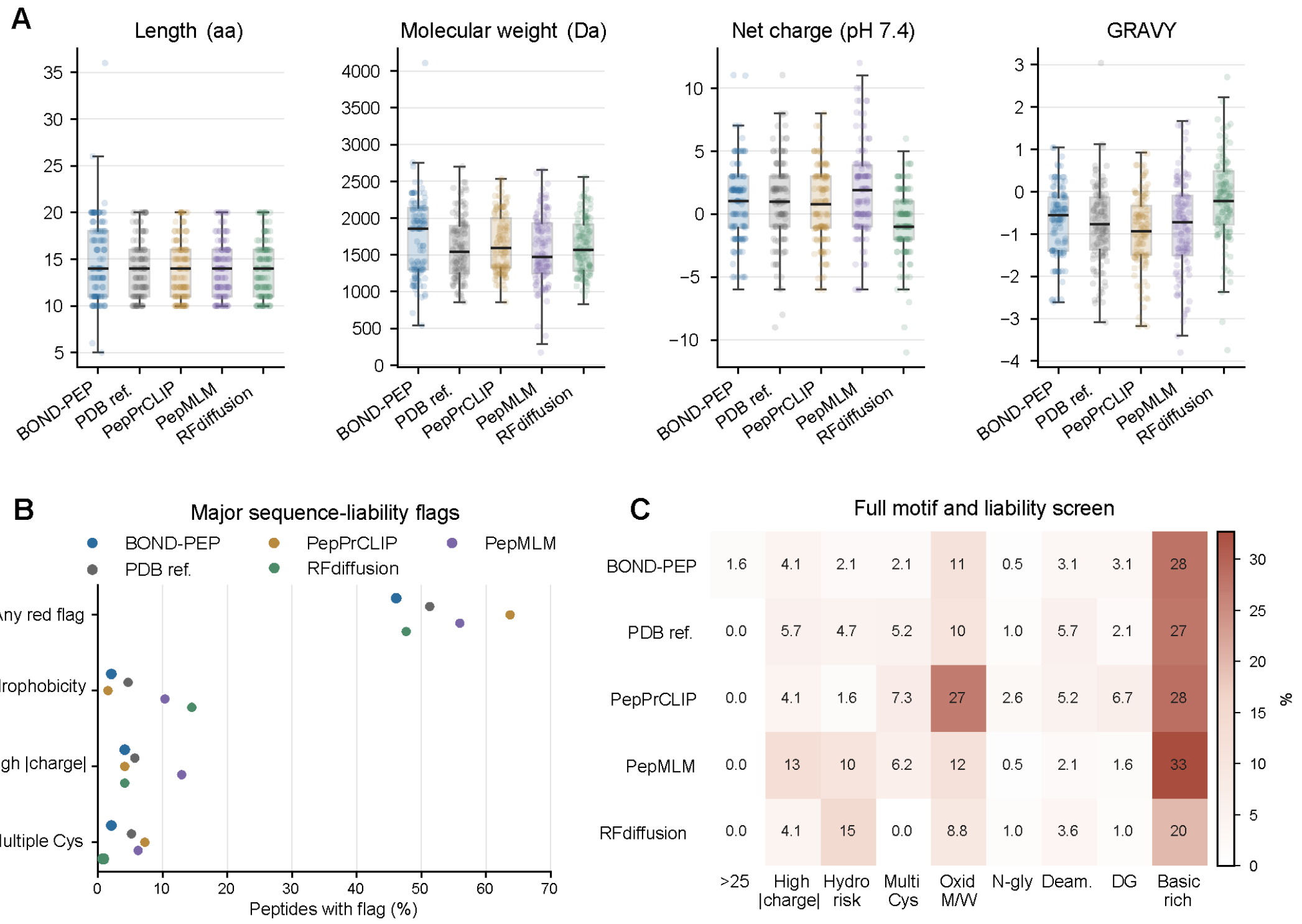

Figure S12. Sequence-level developability-related profiles of generated and reference peptides. Developability-related descriptors were computed on the same 193-target strict held-out benchmark. PDB ref. denotes the corresponding reference peptide. **(A)** Distributions of peptide length, molecular weight, predicted net charge at pH 7.4 and Grand Average of Hydropathy (GRAVY). Boxes show the interquartile range and median; points show individual peptide sequences. **(B)** Rates of the major sequence-liability red flags. Any red flag denotes the overall fraction of peptides carrying at least one developability red flag. **(C)** Full motif-level liability screen across all prespecified red-flag categories.

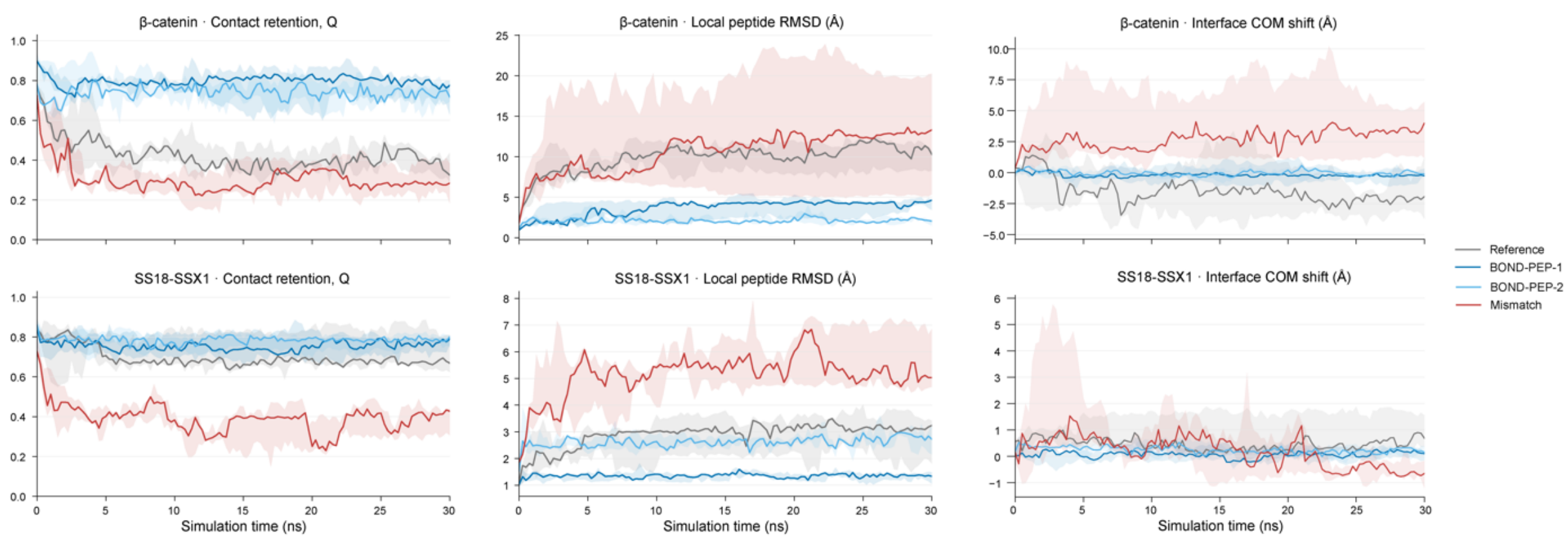

Figure S13. MD time courses of interface retention and peptide displacement. Contact retention Q, local peptide RMSD, and interface COM shift were monitored over 30 ns MD simulations for β-catenin and SS18-SSX1 complexes, with three independent replicas per complex. Lines show replica medians and shaded bands show the replica range. Contact retention Q quantifies retained initial protein-peptide contacts, local peptide RMSD measures binding-pose displacement after protein-interface alignment, and interface COM shift measures peptide center-of-mass drift relative to the initial interface region. Higher Q, lower local peptide RMSD, and lower interface COM shift indicate stronger interface retention.

Supplementary Tables

Table S1. Locality metric summary comparing the original ESM embedding space and the retriever-adapted embedding space. Values are reported as mean ± s.d. across n = 200 independent randomly sampled query targets. The 200 query targets used here are the same locality-analysis subset used in Fig. 3 and are distinct from the 193-pair final benchmark and the external panel. For each query, "top" denotes the top-256 retrieved peptides, whereas "rand" denotes 256 peptides sampled uniformly at random from the same whole peptide library. All values are rounded to four decimal places for consistent presentation and interpretation is unchanged under alternative rounding schemes.

Metric	The original ESM embedding space (mean ± SD)	The retriever-adapted embedding space (mean ± SD)
IntraSim_top	0.9102 ± 0.0365	0.2589 ± 0.1526
IntraSim_rand	0.9349 ± 0.0058	0.0117 ± 0.0036
GtSim_top	0.8615 ± 0.0422	0.4080 ± 0.1334
GtSim_rand	0.9421 ± 0.0213	-0.0034 ± 0.0162
Overlap_top_vs_gtKNN	0.0154 ± 0.0793	0.6679 ± 0.1143
Overlap_rand_vs_gtKNN	0.0353 ± 0.0114	0.0124 ± 0.0078
Seq_intra_JacSim_top	0.0509 ± 0.0672	0.2062 ± 0.2295
Seq_intra_JacSim_rand	0.0033 ± 0.0025	0.0030 ± 0.0019
Seq_gt_JacSim_top	0.0034 ± 0.0171	0.2288 ± 0.2311
Seq_gt_JacSim_rand	0.0048 ± 0.0101	0.0016 ± 0.0027
Seq_intra_BlosumSim_top	-0.0856 ± 0.0870	0.0596 ± 0.3251
Seq_intra_BlosumSim_rand	-0.2838 ± 0.0251	-0.2811 ± 0.0237
Seq_gt_BlosumSim_top	-0.2729 ± 0.0632	0.0956 ± 0.3289
Seq_gt_BlosumSim_rand	-0.3003 ± 0.0820	-0.3066 ± 0.0832

Definitions and computation: All metrics are computed per query and then averaged across queries. "IntraSim" represents within-set similarity, computed as the mean pairwise similarity among peptides within the "top" set or "rand" set in the corresponding representation space. "GtSim" means similarity to the ground-truth, computed as the mean similarity between peptides in the "top" set or "rand" set and the corresponding ground-truth peptide. "Overlap_vs_gtKNN" represents set overlap between the "top" set or "rand" set and the ground truth peptide's KNN set. "Seq_intra_JacSim" and "Seq_gt_JacSim" stand for sequence-level Jaccard similarity computed on peptide sequences under the same "intra-set" and "vs ground-truth" definitions as above. "Seq_intra_BlosumSim" and "Seq_gt_BlosumSim" represent sequence-level similarity computed using a BLOSUM-based scoring scheme under the same "intra-set" and "vs ground-truth" definitions as above. Negative values here indicate lower similarity.

Table S2. Paired statistical tests and confidence intervals for Top vs Random in the original ESM embedding space. We report (a) paired effect sizes and two-sided paired significance tests. (b) reports 95% confidence intervals (CIs) for the mean metric values in each condition. The 200 query targets used here are the same locality-analysis subset used in Fig. 3 and are distinct from the 193-pair final benchmark and the external panel.

(a) Paired tests

Metric	n	Mean (Top-K)	Mean (Random)	Cohen's d	p (Wilcoxon)	p (t-test)
IntraSim	200	0.9102	0.9349	-0.625	1.97e-16	5.06e-16
GtSim	200	0.8615	0.9421	-1.729	3.43e-33	7.50e-62
Overlap	200	0.0154	0.0353	-0.243	3.98e-25	7.16e-04
Seq_intra_JacSim	200	0.0509	0.0033	0.707	1.44e-34	2.58e-19
Seq_gt_JacSim	200	0.0034	0.0048	-0.070	1.29e-02	3.25e-01
Seq_intra_BlosumSim	200	-0.0856	-0.2838	2.164	1.44e-34	3.37e-77
Seq_gt_BlosumSim	200	-0.2729	-0.3003	0.491	9.78e-12	5.20e-11

(b) Confidence intervals

Metric	95% CI (Top-K)	95% CI (Random)
IntraSim	[0.9050, 0.9149]	[0.9341, 0.9357]
GtSim	[0.8557, 0.8673]	[0.9389, 0.9448]
Overlap	[0.0057, 0.0275]	[0.0337, 0.0370]
Seq_intra_JacSim	[0.0430, 0.0614]	[0.0030, 0.0036]
Seq_gt_JacSim	[0.0015, 0.0060]	[0.0035, 0.0063]
Seq_intra_BlosumSim	[-0.0960, -0.0717]	[-0.2873, -0.2803]
Seq_gt_BlosumSim	[-0.2814, -0.2643]	[-0.3125, -0.2896]

Table S3. Paired statistical tests and confidence intervals for Top vs Random in the retriever-adapted embedding space. We report (a) paired effect sizes and two-sided paired significance tests. (b) reports 95% confidence intervals (CIs) for the mean metric values in each condition. The 200 query targets used here are the same locality-analysis subset used in Fig. 3 and are distinct from the 193-pair final benchmark and the external panel.

(a) Paired tests

Metric	n	Mean (Top-K)	Mean (Random)	Cohen's d	p (Wilcoxon)	p (t-test)
IntraSim	200	0.2589	0.0117	1.616	1.44e-34	1.50e-57
GtSim	200	0.4080	-0.0034	3.105	1.44e-34	2.41e-104
Overlap	200	0.6679	0.0124	5.482	1.43e-34	1.21e-150
Seq_intra_JacSim	200	0.2062	0.0030	0.883	1.44e-34	8.22e-27
Seq_gt_JacSim	200	0.2288	0.0016	0.982	3.56e-34	4.09e-31
Seq_intra_BlosumSim	200	0.0596	-0.2811	1.044	2.76e-31	8.64e-34
Seq_gt_BlosumSim	200	0.0956	-0.3066	1.312	1.85e-34	2.83e-45

(b) Confidence intervals

Metric	95% CI (Top-K)	95% CI (Random)
IntraSim	[0.2387, 0.2805]	[0.0111, 0.0122]
GtSim	[0.3898, 0.4271]	[-0.0057, -0.0012]
Overlap	[0.6515, 0.6828]	[0.0113, 0.0135]
Seq_intra_JacSim	[0.1765, 0.2381]	[0.0027, 0.0033]
Seq_gt_JacSim	[0.1981, 0.2624]	[0.0013, 0.0020]
Seq_intra_BlosumSim	[0.0136, 0.1049]	[-0.2845, -0.2778]
Seq_gt_BlosumSim	[0.0516, 0.1404]	[-0.3195, -0.2959]

Table S4. Ablation results at the end of training (70 epochs). We report (a) teacher-forcing and (b) free-generation performance for the full generator ("Full") and three controlled ablations: "Rand" (retrieved top-K context peptides replaced by randomly sampled peptides), "–Topo" (topology conditioner removed), and "Mean" (retrieved peptides aggregated by mean pooling instead of topology bipartite modulation). Results are averaged over five random seeds. Values are mean ± s.d. All settings use the same decoder architecture, training recipe, retrieval library, and identical decoding hyperparameters with the same context budget (K=256 peptides) per target; only the specified component is altered in each ablation. Metrics are computed on the same evaluation split. Uniqueness and Novelty summarize diversity and novelty of the top-8 generated peptides. Exact sequence recovery@8 (ESR@8) indicates whether at least one of the top-8 freely generated peptide candidates exactly matched a reference peptide for the target. ESR mean reciprocal rank@8 (ESR MRR@8) reports the reciprocal rank of the first exact-matching candidate, averaged across targets, with zero assigned when no exact match occurred. Similarity-tolerant sequence recovery@8 (STSR@8) uses the same candidate set but counts a target as recovered when at least one candidate reaches ≥80% sequence identity to a reference peptide. STSR mean reciprocal rank@8 (STSR MRR@8) is computed analogously using the first similarity-tolerant match. Arrows indicate the preferred direction: (↓) stands for lower is better, whereas (↑) means higher is better.

(a) Teacher-forcing metrics

Setting	TF PPL↓	TF Top-5↑
Full	3.452 ± 0.104	0.9052 ± 0.0003
Rand	4.056 ± 0.071	0.8746 ± 0.0005
-Topo	352.47 ± 42.41	0.2992 ± 0.0016
Mean	542.12 ± 65.90	0.2901 ± 0.0020

(b) Free-generation metrics

Setting	ESR@8↑	ESR MRR@8↑	Uniqueness↑	Novelty↑	STSR @8↑	STSR MRR@8↑
Full	0.1989 ± 0.0047	0.1110 ± 0.0067	0.8600 ± 0.0226	0.3386 ± 0.0070	0.3057 ± 0.0065	0.1771 ± 0.0071
Rand	0.1410 ± 0.0023	0.0927 ± 0.0021	0.9376 ± 0.0044	0.0086 ± 0.0018	0.2362 ± 0.0028	0.1540 ± 0.0025
-Topo	0.0000 ± 0.0000	0.0000 ± 0.0000	0.9504 ± 0.0040	1.0000 ± 0.0000	0.0000 ± 0.0000	0.0000 ± 0.0000
Mean	0.0000 ± 0.0000	0.0000 ± 0.0000	0.9398 ± 0.0099	0.9994 ± 0.0005	0.0000 ± 0.0000	0.0000 ± 0.0000

Table S5. Ablation results at the best checkpoints. Same as Table S4, results are averaged over five random seeds. Values are mean ± s.d. All settings use the same decoder architecture, training recipe, retrieval library, and identical decoding hyperparameters with the same context budget per target; only the specified component is altered in each ablation.

(a) Teacher-forcing metrics

Setting	TF PPL↓	TF Top-5↑
Full	3.452 ± 0.104	0.90518 ± 0.00034
Rand	4.278 ± 0.045	0.87678 ± 0.00088
–Topo	23.08 ± 12.23	0.49344 ± 0.04251
Mean	14.402 ± 1.452	0.56238 ± 0.02816

(b) Free-decoding metrics

Setting	ESR@8↑	ESR MRR@8↑	Uniqueness↑	Novelty↑	STSR @8↑	STSR MRR@8↑
Full	0.19886 ± 0.00473	0.1110 ± 0.00667	0.8600 ± 0.02264	0.3386 ± 0.00702	0.30568 ± 0.00650	0.17706 ± 0.00707
Rand	0.16414 ± 0.00182	0.1057 ± 0.00137	0.9236 ± 0.00305	0.0042 ± 0.00045	0.26734 ± 0.00395	0.1722 ± 0.00343
–Topo	0.0013 ± 0.00027	0.00072 ± 0.00024	1.0000 ± 0.0000	0.8808 ± 0.09534	0.0037 ± 0.00146	0.00202 ± 0.00097
Mean	0.00354 ± 0.00118	0.00206 ± 0.00089	1.0000 ± 0.0000	0.6724 ± 0.09834	0.00944 ± 0.00330	0.00488 ± 0.00168

Table S6. Summary of BOND-PEP generated-candidate diversity. For each target, diversity was computed among the BOND-PEP-generated peptide candidates included in the corresponding evaluation. The strictly held-out benchmark used the top-8 generated candidates per target, whereas the external target panel used target-specific candidate numbers matched to the corresponding experimentally tested reference pools.

Dataset	n targets	Mean unique-sequence rate ± S.D.	Median unique-sequence rate	Mean mean pairwise sequence diversity ± S.D.	Median mean pairwise sequence diversity
Held-out strict benchmark	193	0.986 ± 0.075	1.000	0.809 ± 0.072	0.826
External target panel	4	1.000 ± 0.000	1.000	0.796 ± 0.089	0.849

Table S7. Median values of 17 ipTM-independent interface-quality metrics across four peptide designers and reference PDB on the strict held-out benchmark. Arrows indicate favorable direction: ↑, higher is better; ↓, lower is better.

Metric (unit; favorable direction)	BOND-PEP	Reference	PepMLM	PepPrCLIP	RFdiffusion
Buried SASA (Å²; ↑)	1267.6	1237.3	1255.8	1259.6	1194.0
Atom contacts (count; ↑)	237	236	244	280	215
Residue contacts (count; ↑)	38	36	37	36	33
Peptide-contact fraction (fraction; ↑)	0.727	0.769	0.778	0.750	0.750
Contact density (atom contacts / 100 Å²; ↑)	17.8	18.8	18.9	21.9	18.0
Geometry H-bonds (atom pairs; ↑)	10	11	11	13	9
Salt bridges (atom pairs; ↑)	2	2	2	1	2
Hydrophobic pairs (residue pairs; ↑)	11	11	11	10	12
Unsat. H-bonds (count; ↓)	9	11	12	16	10
Rosetta interface H-bonds (count; ↑)	4	3	3	2	1
Steric clashes (atom pairs; ↓)	1	1	2	8	3
Rosetta packstat (score; ↑)	0.626	0.622	0.615	0.627	0.601
Shape complementarity (score; ↑)	0.672	0.652	0.611	0.422	0.542
Rosetta ΔG separated (REU; ↓)	464.4	522.6	729.8	2598.9	1020.0
Rosetta ΔSASA (Å²; ↑)	1331.2	1312.1	1324.7	1368.6	1265.1
Hydrophobic ΔSASA (Å²; ↑)	830.8	783.9	832.4	816.4	813.6
Polar ΔSASA (Å²; ↑)	531.4	508.1	516.2	525.5	442.2

Table S8. Comparison of BOND-PEP with experimentally validated peptides. For each target, the reference pool consisted of the corresponding source-study reported peptide candidates: 6 for β-catenin, 20 for UltraID, 10 for SS18-SSX1 and 4 for NCAM1. The peptide with the highest candidate-level ipTM was recorded as the reference entry. BOND-PEP was evaluated using the same target-specific budget for each target, and the BOND-PEP peptide with the highest candidate-level ipTM was reported here.

Target name	Reference method	Best validated peptide	ipTM (reference)	BOND-PEP peptide	ipTM (BOND-PEP)
β-catenin	PepPrCLIP	EESDDLYIP AFLRKQLD	0.486	VEPQKFAEE LIHRLEAVQ	0.871
UltraID	PepPrCLIP	DNNHCSWL YGYWEWTL WGED	0.356	CYIQNCPLG	0.563
SS18-SSX1	PepPrCLIP	LWWGMAK YCAAKLTC MC	0.551	RIIYDRKFL MECRN	0.874
NCAM1	PepMLM	GLGPSPVLP RC	0.571	EPQKFAEEL IHRLEAVQ	0.709

Supplementary Methods

PLMs evaluation

Evolutionary Scale Modeling (ESM) is a series of transformer protein language models developed by Meta AI's FAIR team. Among them, ESM-2 (released in 2022) has become one of the most widely used PLMs in protein informatics. At the end of 2024, EvolutionaryScale introduced ESM-3 and ESM Cambrian (ESM-C) as parallel model families. While ESM-3 is designed as a protein generative model, ESM-C is oriented toward sequence representation learning aimed at underlying biology. Here, we evaluated ESM-2-650M and ESM-C-600M under identical preprocessing. Both models used the same standard amino-acid tokenizer, and we used the model's native output logits directly, ensuring our metrics reflect each model's intrinsic residue-level predictive distribution properly.

To provide an interpretable and reasonably balanced presentation scheme, we stratified sequences into proteins and peptides grouped by length (>15, 11–15, and ≤10 amino acids), and then randomly sampled 1000 sequences for each category from our sequence dataset. To evaluate their performance gap between proteins and peptides, we ran ESM-2-650M and ESM-C-600M on three complementary tasks: self-copy, LOO and denoising.

For self-copy, the full unmasked sequence $s = (y_1, \ldots, y_L)$ was provided as input, and the model produced logits at each position. We computed per-position Top-k correctness by checking whether the true amino acid y_i appeared among the k highest-probability tokens at position i. We reported Top-1 accuracy in our results. Sequence-level accuracy was the mean across positions as follows:

$$\mathrm{Acc@k}(s) = \frac{1}{L}\sum_{i=1}^{L} \mathbb{1}\{\, y_i \in \mathrm{TopK}(\hat{p}_i)\}.$$

For LOO, we evaluated each position independently by masking position i and predicting y_i from the remaining context. LOO task is more stringent than self-copy, and we reported not only Top-1 accuracy but also pseudo-perplexity (pPPL) to quantify overall predictive uncertainty. pPPL is derived from the negative log-likelihood (NLL). Averaging NLL over positions gives the typical "surprisal" of native residues from a sequence. Exponentiating this quantity yields pPPL, which can be interpreted as the model's effective number of competing choices per position. Lower pPPL means less uncertainty, which further means the better model performance. We computed pPPL via:

$$\mathrm{pPPL}(s) = \exp\{\frac{1}{L}\sum_{i=1}^{L} -\log\ p(y_i \mid x_i)\}.$$

For denoising, we first corrupted the sequence by randomly replacing 20% residues and then iteratively refined the sequence for n steps. n equals the number of replaced residues. For each refinement step, the lowest-confidence position under the current model prediction was identified and then resampled via masked inference. We used Hamming similarity to quantify the reconstruction quality between the final sequence $\hat{s}$ and the ground truth s as follows:

$$\mathrm{HamSim}(s, \hat{s}) = \frac{1}{L}\sum_{i=1}^{L} \mathbb{1}\{\, \hat{y}_i = y_i\}.$$

To test whether the length-associated PLM trend depended on the original three-bin (>15, 11–15, and ≤10 amino acids) presentation, we performed three cutoff-independent analyses. First, peptide length was treated as a continuous variable, and its association with LOO Top-1 recovery, LOO Top-5 recovery, LOO pseudo-perplexity, and denoising metrics was quantified using Spearman correlation. Bootstrap 95% confidence intervals were estimated using 1000 resamples. Second, peptides were regrouped into data-adaptive length quartiles, avoiding manually chosen cutoffs. Third, we exhaustively scanned all valid two-cutpoint partitions of the 1000-peptide set, requiring at least 100 peptides per bin, and recorded whether each partition supported the same short-worse direction for recovery and uncertainty.

To evaluate the contribution of reduced sequence context to the PLM degradation observed for peptides, we generated a length-matched protein-fragment control. For each peptide in the 1000-peptide PLM evaluation set, we sampled a protein fragment with the same length from the sampled full-length protein pool. These length-matched protein fragments were then evaluated using the same preprocessing, ESM-2-650M and ESM-C-600M models, and PLM scoring functions as used before.

To assess whether the low ESM-C self-copy Top-1 recovery reflected an implementation issue, we performed a series of sanity checks. For example, the tokenizer produced the expected "<cls> sequence <eos>" format with logits length L+2, residue i corresponded to logits row 1+i and all 20 canonical amino-acid tokens were correctly mapped. All our implementation strictly followed the ESM-C official documentation and all the intermediate outputs were consistent with the documented API behavior. We also compared raw Top-1 recovery with amino-acid-restricted Top-1 recovery to test whether special-token or non-amino-acid predictions explained the low Top-1 self-copy value. These checks did not identify a tokenizer, offset, amino-acid-vocabulary, or special-token artifact.

Precomputed token embeddings

All proteins and peptides were represented using precomputed token-level embeddings from ESM-2-650M, stored on CPU to minimize GPU memory usage. Specifically, we stored: protein embeddings $X^{(prot)} \in \mathbb{R}^{N_{\text{prot}} \times L_{\text{prot}}^{\text{max}} \times \text{F}}$ and corresponding masks $M^{(prot)} \in \{0,1\}^{N_{\text{prot}} \times L_{\text{prot}}^{\text{max}}}$; peptide embeddings $X^{(pep)} \in \mathbb{R}^{N_{\text{pep}} \times L_{\text{pep}}^{\text{max}} \times \text{F}}$ and corresponding masks $M^{(pep)} \in \{0,1\}^{N_{\text{pep}} \times L_{\text{pep}}^{\text{max}}}$. Here $L_{\text{prot}}^{\text{max}}$ and $L_{\text{pep}}^{\text{max}}$ denote the maximum sequence length within the protein and peptide libraries. We padded proteins and peptides to these fixed max lengths respectively and indicated padded positions by mask value 0. F is the per-token embedding dimension produced by ESM-2-650M. It is worth noting that during network computation, only the embeddings required for a minibatch were transferred to GPU using non-blocking copies.

We treated the ESM special BOS token differently for proteins and peptides. For peptides, we discarded BOS, thus each peptide mask contains exactly L ones corresponding to residues. In contrast, for proteins we retained BOS, resulting in $1 + L$ valid positions per protein sequence. This choice was determined by strictly controlled comparison in which we evaluated models trained with and without BOS token for each modality and selected the configuration that yielded the best performance. We hypothesize that BOS is less informative for short peptides, consistent with the degradation of peptide representations observed in our previous analyses, whereas for full-length proteins the BOS token captures useful global context.

Sequence encoders

We use an attention module called AttnSeqEncoder as our protein encoder. Given a batch of ESM protein token embeddings $X_b^{(prot)} \in \mathbb{R}^{\mathrm{b} \times L_{\mathrm{prot}}^{\max} \times \mathrm{F}}$ and corresponding masks, we first project each token to a hidden representation: $h_t = f_{\mathrm{tok}}(x_t) \in \mathbb{R}^d$, where f_{tok} is a multi-layer perceptron (MLP) with layer normalization and dropout. From this, we compute scalar attention logits using: $e_t = w^\top \tanh(W h_t) \in \mathbb{R}$. We mask invalid tokens by setting logits to $-\infty$, and thus form normalized attention weights:

$$\alpha_t = \frac{\exp\,(e_t)}{\sum_{u:\,M_u=1} \exp\,(\,e_u)}.$$

Finally, the pooled protein embedding is obtained by applying a linear head to the attention-weighted sum of token representations, yielding a D-dimensional vector (default $D = 512$):

$$\mathrm{prot\_init}_b = W_{prot\_init}\left(\sum_{t=1}^{\mathrm{L}} \alpha_t\, h_t\right) \in \mathbb{R}^{b \times D}.$$

Analogously, peptide sequences are encoded with a convolutional module called ConvSeqEncoder. First, a batch of ESM token embeddings of peptides $X_b^{(pep)} \in \mathbb{R}^{\mathrm{b} \times L_{\mathrm{pep}}^{\max} \times \mathrm{F}}$ with corresponding masks are transposed and projected into a model width d_{model}. Then a stack of residual 1D convolution blocks are applied as:

$$H^{(\ell+1)} = H^{(\ell)} + \mathrm{Dropout}\left(\mathrm{GELU}\left(\mathrm{GroupNorm}\left(\mathrm{Conv1D}(H^{(\ell)})\right)\right)\right).$$

The final pooled peptide embedding is computed by applying a linear head to the masked mean pooling:

$$\mathrm{pep\_init}_b = W_{pep\_init}\left(\frac{\sum_t M_t\ H_t}{\sum_t M_t}\right) \in \mathbb{R}^{b \times D},$$

Retriever architecture

The retriever is implemented as an independent dual-encoder module that embeds proteins and peptides separately into a shared retrieval space, enabling efficient maximum-inner-product search for any query protein over large peptide libraries. Given token-level embeddings and masks, the protein tower (AttnSeqEncoder) and the peptide tower (ConvSeqEncoder) produce fixed-dimensional representations $\mathrm{prot\_init}, \mathrm{pep\_init} \in \mathbb{R}^D$, which are then mapped into the shared retrieval space by modality-specific projection heads:

$$z^{(prot)} = g_{prot}(\mathrm{prot\_init}) \in \mathbb{R}^{d_r}, z^{(pep)} = g_{pep}(\mathrm{pep\_init}) \in \mathbb{R}^{d_r},$$

where $d_r = 2048$ is the dimension of the retrieval space. Both projection heads $g_{prot}(\cdot)$ and $g_{pep}(\cdot)$ are implemented as residual MLPs with LayerNorm and Dropout:

$$g(x) = W_{g2}\ \mathrm{Dropout}\left(\mathrm{GELU}\left(W_{g1}\ \mathrm{LN}(x)\right)\right) + W_{\text{g-skip}}\ \mathrm{LN}(x).$$

This design improves optimization stability compared to a single linear projection, while preserving a direct path from normalized base embeddings to the retrieval space.

Candidate peptides are then ranked by dot-product similarity:

$$s(prot, pep) = \langle z^{(prot)}, z^{(pep)} \rangle.$$

This is equivalent to cosine similarity when using embedding normalization. The factorized scoring allows offline precomputation of peptide embeddings and scalable top-K retrieval without cross-attention between modalities. To stabilize similarity score $s(prot, pep)$ in the high-dimensional retrieval space, we normalize vectors using a multi-subspace scheme. More specifically, for any projected vector $z \in \mathbb{R}^{d_r}$, we reshape it into M sub-vectors $z^{(m)} \in \mathbb{R}^{d_h}$ where $d_h = d_r/M$, then normalize each sub-vector independently:

$$\hat{z}^{(m)} = \frac{z^{(m)}}{\| z^{(m)} \|_2} .$$

We further introduce learnable head importances via logits $\gamma \in \mathbb{R}^M$, forming weights

$$w_m = M \cdot \mathrm{softmax}(\gamma)_m,$$

and apply them as $\sqrt{w_m}$ scaling:

$$\tilde{z}^{(m)} = \sqrt{w_m}\, \hat{z}^{(m)}.$$

Finally, we scale by $1/\sqrt{M}$ and concatenate:

$$\tilde{z} = \frac{1}{\sqrt{M}} [\tilde{z}^{(1)}; \dots; \tilde{z}^{(M)}] \in \mathbb{R}^{d_r}.$$

With this construction, the dot product decomposes into a weighted sum of per-subspace cosines:

$$\left\langle \tilde{z}_{prot}, \tilde{z}_{pep} \right\rangle = \frac{1}{M} \sum_{m=1}^{M} w_m \cos\left(z_{prot}^{(m)}, z_{pep}^{(m)}\right).$$

This multi-subspace or multi-head scheme yields stable magnitudes and allows the model to learn which subspaces are more informative for retrieval.

Retriever pretraining

We pretrain the retriever using a two-stage contrastive objective. Specifically, we optimize the retriever encoders, projection heads, and head-importance logits γ by minimizing a weighted sum of an in-batch contrastive loss and an auxiliary hard-negative mining loss:

$$\mathcal{L}_{\mathrm{retr}} = \lambda_{\mathrm{ctrs}} \mathcal{L}_{\mathrm{ctrs}} + \lambda_{\mathrm{hard}} \mathcal{L}_{\mathrm{hard}},$$

with $\lambda_{\mathrm{ctrs}} = 1.0$ and $\lambda_{\mathrm{hard}} = 0.3$. To make the training stable, hard-negative mining is enabled only after the 12th epoch.

Given a minibatch of b (default batch size b is 512) matched protein–peptide pairs $\{(\mathrm{prot}_i, \mathrm{pep}_i)\}_{i=1}^{b}$, we compute normalized retrieval vectors $\tilde{z}_i^{(\mathrm{prot})}$ and $\tilde{z}_i^{(\mathrm{pep})}$ as described above, and then form a temperature-scaled similarity matrix:

$$S_{ij} = \frac{\langle \tilde{z}_i^{(\mathrm{prot})}, \tilde{z}_j^{(\mathrm{pep})} \rangle}{\tau}, \qquad \tau = 0.07.$$

Using the paired examples (i, i) as positives and all off-diagonal entries as in-batch negatives, we minimize a cross-entropy loss for protein→peptide retrieval:

$$\mathcal{L}_{\text{prot}\rightarrow\text{pep}} = -\frac{1}{b}\sum_{i=1}^{b}\log\frac{\exp(S_{ii})}{\sum_{j=1}^{b}\exp(S_{ij})}.$$

To avoid modality-specific collapse and enforce bidirectional alignment, we use the symmetric variant by adding the reverse direction, *i.e.*, peptide→protein retrieval:

$$\mathcal{L}_{\text{pep}\rightarrow\text{prot}} = -\frac{1}{b}\sum_{j=1}^{b}\log\frac{\exp(S_{jj})}{\sum_{i=1}^{b}\exp(S_{ij})}, \qquad \mathcal{L}_{\text{ctrs}} = \frac{1}{2}\left(\mathcal{L}_{\text{prot}\rightarrow\text{pep}}+\mathcal{L}_{\text{pep}\rightarrow\text{prot}}\right).$$

To further sharpen the retrieval beyond in-batch negatives, we additionally optimize a hard-negative objective after the 12th epoch. At initialization and after every epoch, we refresh a CPU-resident peptide index by encoding the full peptide library with the current retriever parameters to obtain a normalized retrieval matrix

$$\tilde{Z}_{\text{pep}} = \left[\tilde{z}_1^{(\text{pep})}; \ldots; \tilde{z}_{N_{\text{pep}}}^{(\text{pep})}\right] \in \mathbb{R}^{N_{\text{pep}}\times d_r}.$$

For a subset of query proteins per training step, we retrieve 2048 peptides by dot-product search against $\tilde{Z}_{\text{pep}}$. To reduce ambiguity from near-duplicates or extremely similar candidates, we discard the highest-ranked 64 retrieved peptides and select the subsequent 256 candidates as hard negatives $\mathcal{N}_i$. We then increase separation between the positive peptide and the mined negatives using a contrastive loss of the following form:

$$\mathcal{L}_{\text{hard}} = -\frac{1}{|Q|}\sum_{i\in Q}\log\frac{\exp\left(\langle\tilde{z}_i^{(\text{prot})},\tilde{z}_i^{(\text{pep})}\rangle/\tau_i\right)}{\exp\left(\langle\tilde{z}_i^{(\text{prot})},\tilde{z}_i^{(\text{pep})}\rangle/\tau_i\right)+\sum_{j\in\mathcal{N}_i}\exp\left(\langle\tilde{z}_i^{(\text{prot})},\tilde{z}_j^{(\text{pep})}\rangle/\tau_i\right)},$$

where $\tau_i = \max(\tau, \tau_{\min})$ applies a temperature floor $\tau_{\min} = \text{hard\_temp\_floor} = 0.1$ for numerical stability. This auxiliary term explicitly trains the retriever to down-rank highly confounding peptides that are close to the query in the current embedding space.

We optimize retriever parameters using AdamW with learning rate $\text{lr} = 4 \times 10^{-4}$ and weight decay $\text{weight\_decay} = 10^{-4}$. A stepwise learning-rate schedule is applied. To select retriever checkpoints robustly, we performed 5-fold cross-validation with shuffling. For evaluation, we compute Recall@ K for multiple K values (128, 256, 512 and 1024) on the held-out fold:

$$\text{Recall@}K = \frac{1}{N}\sum_{n=1}^{N}\mathbb{1}\left[\text{pep}_n \in \text{Top}K(\text{prot}_n)\right].$$

We also monitor the retriever via coverage of the peptide library defined as the fraction of unique peptides appearing in retrieved lists:

$$\text{Coverage@K} = \frac{\left|\bigcup_n \text{TopmaxK}(\text{prot}_n)\right|}{N_{\text{pep}}}.$$

ESM-embedding-space distribution analysis

To assess distributional differences between ESM representations of proteins and peptides, we randomly drew 2,000 protein–peptide pairs from our dataset. For each protein and peptide, variable-length ESM embeddings were converted to fixed-dimensional vectors by mean pooling. We first projected pooled embeddings to one dimension using principal component analysis (PCA) and then visualized distributional differences via kernel density

estimation (KDE). Specifically, we estimated the marginal density of the first principal component for proteins and peptides separately using Gaussian KDE and plotted the resulting density curves as filled overlays. Density curves are normalized such that the area under each curve integrates to 1.

Candidate-set level assessment of peptide-space

We performed a candidate-set level analysis on $N_q = 200$ randomly sampled proteins to probe how peptide representations differ between the original ESM embedding space and the retriever-adapted embedding space. For each protein query, we constructed a top-ranked candidate set under each space and compared it to a size-matched random baseline using paired measurements (top vs random for the same query). This design allowed us to quantify how each embedding space shapes (i) within-set cohesion, (ii) proximity to the ground-truth (GT) peptide, and (iii) overlap with the GT peptide's local neighbourhood. We also did complementary sequence-based analyses to test whether candidates selected in the native ESM space versus the retriever-adapted space differ at the sequence level, including k-mer Jaccard and BLOSUM62 alignment similarity as a proxy for amino-acid substitution and biochemical property similarity.

More specifically, each entry is a tuple (p_{gt}, t) specifying a protein indexed t and its associated GT peptide indexed p_{gt} with amino-acid sequence string $s_{p_{gt}}$. For each query protein t, we constructed two candidate peptide sets of size $\mathrm{K} = 256$ from peptide library $p \in \mathcal{P}$ in parallel: top candidate set C_t^{top} and random baseline C_t^{rand} for both embedding spaces. For ESM embedding space, we first obtained protein and peptide representations q_t^{ESM} and x_p^{ESM} by mean pooling of ESM embeddings and then performed retrieval as:

$$C_t^{\text{top,ESM}} = \text{TopK}_{\,p \in \mathcal{P}} \cos\left(q_t^{\text{ESM}}, x_p^{\text{ESM}}\right).$$

Meanwhile, for retriever-adapted embedding space, we loaded a pre-trained retriever checkpoint to compute representations of query protein h_t^{Retr} and peptide library $\{z_p^{Retr}\}$. The retrieval was then performed by the retriever's scoring function, yielding $C_t^{top,Retr}$. Their random baseline $C_t^{rand,ESM}$ and $C_t^{rand,Retr}$ were sampled uniformly without replacement from the peptide library in corresponding embedding spaces.

All embedding-space metrics used cosine similarity. For each query (p_{gt}, t), we computed the following metrics for both C_t^{top} and C_t^{rand} across all embedding spaces:
(1) Candidate-set intra-similarity (within-set cohesion):

$$S_{\text{intra}}(C) = \mathbb{E}_{p_i \neq p_j,\; p_i, p_j \in C}[\cos\,(\mathrm{x}_{p_i}, \mathrm{x}_{p_j})].$$

We approximated this expectation by sampling 4000 random ordered pairs (i, j) with $i \neq j$ per query.
(2) Similarity of candidates to the GT peptide:

$$S_{\text{gt}}(C) = \frac{1}{\mid C \mid} \sum_{p \in C} \cos\,(\mathrm{x}_p, \mathrm{x}_{p_{gt}}).$$

(3) Overlap with the GT embedding neighbourhood:
We first computed the GT peptide's K-nearest neighbours in the same embedding space across the full peptide library:

$$\mathrm{NN_K}(p_{gt}) = \mathrm{TopK}_{\,p\in\mathcal{P}} \cos\,(\mathrm{x}_p, \mathrm{x}_{p_{gt}}).$$

We then computed the overlap fraction:

$$O(C) = \frac{|\, C \cap \mathrm{NN_K}(p_{gt}) \,|}{\mathrm{K}},$$

which captures whether the candidate set is enriched for peptides in the immediate embedding neighbourhood of the GT peptide.

We also did complementary sequence-based analyses. Related sequence-space metrics are:
(4) k-mer Jaccard similarity ($k = 3$):
Given a peptide sequence s, we represent it by the set of all overlapping k-mers, denoted $\mathcal{K}_k(s)$. For two sequences a and b, their k-mer Jaccard similarity is defined as:

$$J_k(a,b) = \frac{|\, \mathcal{K}_k(a) \cap \mathcal{K}_k(b) \,|}{|\, \mathcal{K}_k(a) \cup \mathcal{K}_k(b) \,|}.$$

We then computed intra-set similarity and GT similarity of peptide sequences like (1) and (2).

(5) BLOSUM62 alignment-based similarity:
We computed global alignment scores under BLOSUM62 substitution scores using a Needleman–Wunsch dynamic program with affine gap penalties:

$$g(l) = g_{\mathrm{open}} + (l-1)\, g_{\mathrm{extend}},$$

where $g(\ell)$ is the total penalty for a contiguous gap of length ℓ, g_{open} penalizes opening a new gap, and g_{extend} penalizes extending an existing gap by one position. We set $g_{\mathrm{open}} = -11$ and $g_{\mathrm{extend}} = -1$. For sequences a, b, let $S(a,b)$ be the optimal global alignment score. To normalize for length and composition, we used:

$$\tilde{S}(a,b) = \frac{S(a,b)}{\frac{1}{2}(S(a,a) + S(b,b))}.$$

Similarly, intra-set similarity and GT similarity was computed like (4) as well.

For each metric m, we obtained paired per-entry measurements $\{m_i^{top}, m_i^{rand}\}_{i=1}^{N_q}$ and analysed paired differences $\Delta_i = m_i^{top} - m_i^{rand}$. We assessed significance using two-sided Wilcoxon signed-rank test and paired t-test, quantifying effect size using paired Cohen's $d = \mathrm{mean}(\Delta)/\mathrm{sd}(\Delta)$.

Retriever freezing and peptide library preparation

Once the retriever training is completed, we load the pretrained retriever checkpoint and freeze all retriever parameters ($\nabla_{\theta_{\mathrm{retriever}}}\mathcal{L} = 0$), ensuring retrieval behavior remains fixed during all subsequent training and inference stages. To accelerate training and inference, we precompute retrieval embeddings for the entire peptide library. First, the peptide tower produces base embeddings $\mathrm{pep\_init}_i \in \mathbb{R}^D$ for all peptides $i \in \{1, \ldots, N_{\mathrm{pep}}\}$. Then the projection head and multi-subspace normalization produce retrieval embeddings $\tilde{z}_i^{(pep)} \in \mathbb{R}^{d_r}$. The resulting library matrix is as follows:

$$Z_{\text{pep}} = \begin{bmatrix} \tilde{z}_1^{(pep)} \\ \vdots \\ \tilde{z}_{N_{\text{pep}}}^{(pep)} \end{bmatrix} \in \mathbb{R}^{N_{\text{pep}} \times d_r},$$

is stored on CPU to minimize GPU memory usage.

For each query protein, we compute the protein retrieval embedding $\tilde{z}^{(prot\_query)}$ from the protein tower output and projection head. We then compute similarities by multiplication:

$$\mathbf{s} = Z_{\text{pep}}\, \tilde{z}^{(prot\_query)} \in \mathbb{R}^{N_{\text{pep}}},$$

and select the indices of largest 256 scores. These indices define the candidate peptide set used downstream for topology conditioning and sequence generation.

Topology conditioner

Topology conditioning is performed via a local bipartite subgraph per protein. For each minibatch of size b, we construct this protein–peptide local bipartite graph $G = (V, E)$ that couples each query protein to its retrieved top-K (default $K = 256$) peptide context. The node set is defined as:

$$V = V_{\text{prot}} \cup V_{\text{pep}}, V_{\text{prot}} = \{1, \dots, b\}, V_{\text{pep}} = \{\, (n, k) \mid n \in [1, b], k \in [1, K] \,\}.$$

We instantiate two directed edge sets that form a star-shaped subgraph for each protein:

$$E_{\text{prot}\to\text{pep}} = \{\, n \to (n, k) \,\}_{n,k}, \qquad E_{\text{pep}\to\text{prot}} = \{\, (n, k) \to n \,\}_{n,k}.$$

Thus, the graph contains exactly bK directed edges in each direction and no cross-protein edges among peptide candidates. Initial node features are given by the batch protein embedding $\text{prot\_init}_n \in \mathbb{R}^D$ and the retrieved peptide embeddings $\text{pep\_init\_sub}_{n,k} \in \mathbb{R}^D$.

Topology conditioning is performed using L (default $L = 3$) stacked multi-head bipartite graph-attention layers (called MultiHeadBiGAT module). Each layer contains two directional multi-head attention blocks with independent parameters: (i) a protein→peptide update that aggregates protein information into each peptide node, and (ii) a peptide→protein update that aggregates peptide information into each protein node. For clarity, let H denote the number of attention heads (default $H = 4$). For a generic directed update from source node i to destination node j, we apply pre-normalization (LayerNorm) and head-specific linear projections:

$$s_i^{(h)} = W_s^{(h)}\, \text{LN}(h_i), \qquad d_j^{(h)} = W_d^{(h)}\, \text{LN}(h_j), \qquad h \in \{1, \dots, H\},$$

where s represents source and d for destination. Attention logits follow the additive form implemented in MultiHeadGAT and we also add a cosine-similarity bonus. Thus, the unnormalized edge score would be:

$$e_{ij}^{(h)} = u^{(h)\top}\, \text{LeakyReLU}\left(s_i^{(h)} + d_j^{(h)}\right) + \exp(\eta) \cdot \cos\left(s_i^{(h)}, d_j^{(h)}\right),$$

where $u^{(h)}$ is a learnable head-specific vector and η is a learnable scalar. Normalized attention coefficients are computed for each destination node using a stable segment-wise softmax over its incoming edges followed by attention dropout:

$$\alpha_{ij}^{(h)} = \frac{\exp\,(e_{ij}^{(h)})}{\sum_{i' \in \mathcal{N}(j)} \exp\,(e_{i'j}^{(h)})}, \qquad i \in \mathcal{N}(j) \ .$$

Messages are aggregated per head as a weighted sum of source projections:

$$m_j^{(h)} = \sum_{i \in \mathcal{N}(j)} \alpha_{ij}^{(h)} \ s_i^{(h)}.$$

Head outputs are merged either by mean or concatenation, and then projected to the output width and combined with a residual connection from the destination state as follows:

$$\widetilde{m}_j = \mathrm{Merge}(\{m_j^{(h)}\}_{h=1}^{H}), \qquad h_j' = ReLU\big(W_{\mathrm{merge}} \widetilde{m}_j + W_{\mathrm{res}} h_j\big) \ .$$

Applying this operator to both directions yields one bipartite layer update:

$$h_{\mathrm{pep}}^{(\ell+1)} = \mathrm{GAT}_{\mathrm{prot}\to\mathrm{pep}}\Big(h_{\mathrm{prot}}^{(\ell)}, h_{\mathrm{pep}}^{(\ell)}, E_{\mathrm{prot}\to\mathrm{pep}}\Big),$$
$$h_{\mathrm{prot}}^{(\ell+1)} = \mathrm{GAT}_{\mathrm{pep}\to\mathrm{prot}}\Big(h_{\mathrm{pep}}^{(\ell)}, h_{\mathrm{prot}}^{(\ell)}, E_{\mathrm{pep}\to\mathrm{prot}}\Big),$$

and we have L stacked layers as mentioned before.

After L bipartite layers, we take the updated protein node state as the final topology-conditioned vector. Notably, the vector remains protein-centric, while being selectively modulated by retrieved peptides through the peptide→protein attention. This attention mechanism learns to weight and integrate information across the top-K candidate peptides for each query protein.

During generation, we optionally expand the retrieved candidate pool to top-Mpeptides ($M \geq K$) and randomly subsample K peptides multiple times to construct several stochastic local subgraphs per protein. This yields multiple topology-conditioned vectors that correspond to different retrieved-context views.

Peptide generator

We generate peptides over an amino-acid vocabulary $A = \{$20 canonical residues$\}$ augmented with special tokens: $V = A \cup \{$BOS, EOS, PAD, UNK, MASK$\}$, where BOS and EOS delimit sequences, PAD denotes padding, UNK denotes out-of-vocabulary residues, and MASK is used only by the auxiliary masked objective. For a peptide $s = (s_1, \dots, s_L)$, we construct teacher-forcing inputs and targets as

$$x = (\mathrm{BOS}, s_1, \dots, s_L), \qquad y = (s_1, \dots, s_L, \mathrm{EOS}).$$

We use a Transformer decoder with N_D layers (default 4) and H_D attention heads (default 8). The model width of the decoder is d_{model} (default 512) and it contains dropout (default rate 0.1). The topology-conditioned vector of query protein mentioned above, $\mathrm{prot\_topo} \in \mathbb{R}^D$ is projected into the decoder width and injected as a single memory token:

$$\mathrm{mem} = W_c \ \mathrm{prot\_topo} \in \mathbb{R}^{1 \times b \times d_{\mathrm{model}}},$$

where b is the minibatch size. Token embeddings and learned positional embeddings are summed as $u_t = E(x_t) + P(t)$ for position t followed by dropout. The decoder computes hidden states o_t and outputs vocabulary logits:

$$\ell_t = W_o\, o_t \in \mathbb{R}^{|V|},$$

For autoregressive decoding, we apply a standard causal self-attention mask so that each position attends only to $\leq t$. We use teacher forcing with cross-entropy over all non-padding positions for primary training:

$$\mathcal{L}_{\text{AR}} = -\sum_{n=1}^{b} \sum_{t=1}^{T} \mathbb{1}\left[y_{n,t} \neq \text{PAD} \right] \log\, p\left(y_{n,t} \mid x_{n,\leq t}, \text{prot\_topo}_n \right).$$

To encourage global consistency while preserving the autoregressive interface, we add an auxiliary Span-MLM objective. For each input x, we sample a set of masked positions $M \subseteq \{1, \ldots, T\}$ by selecting contiguous spans until reaching an expected masked fraction p_{mlm} (default 0.15), excluding BOS and any PAD positions. Span lengths L_s are drawn from a geometric distribution with mean μ (default 3) and truncated at $L_{\max}$ (default 10). At masked positions, we apply a BERT-style replacement rule: 80% replaced by MASK, 10% replaced by a random amino acid, and 10% left unchanged, yielding a corrupted input $\tilde{x}$. We define MLM labels $\tilde{y}$ as

$$\tilde{y}_t = \begin{cases} x_t, & t \in M, \\ -100, & t \notin M, \end{cases}$$

where -100 corresponds to ignored targets in the cross-entropy implementation.

A naïve MLM pass would allow bidirectional attention for all tokens, which encourages reliance on "future" context and thus degrade left-to-right generation. We therefore use a selective bidirectional mask: unmasked query positions remain causal, while masked query positions may attend to the full context. Formally, for a query position t, allowed keys u would satisfy:

$$u \leq t \ \text{ if } t \notin M, \qquad u \in \{1, \ldots, T\} \ \text{ if } t \in M.$$

Equivalently, the boolean attention mask $A \in \{0,1\}^{T \times T}$ (1 indicates forbidden) is:

$$A_{t,u} = \begin{cases} 1, & (u > t) \wedge (t \notin M), \\ 0, & \text{otherwise.} \end{cases}$$

The Span-MLM forward pass uses the same conditioning memory while replacing the standard causal mask with A, and we compute cross-entropy only at masked positions as follows:

$$\mathcal{L}_{\text{MLM}} = -\sum_{n=1}^{b} \sum_{t=1}^{T} \mathbb{1}\left[t \in M_n \right] \log\, p\left(x_{n,t} \mid \tilde{x}_n, \text{prot\_topo}_n;\, A \right).$$

The final objective combines autoregressive and auxiliary losses:

$$\mathcal{L} = \mathcal{L}_{\text{AR}} + \lambda\, \mathcal{L}_{\text{MLM}}.$$

To avoid early optimization instability, we linearly warm up the MLM weight during the first E_w epochs:

$$\lambda(e) = \lambda_{\max} \cdot \min\left(1, \frac{e}{E_w}\right),$$

where e is the epoch index, and the default of $\lambda_{\max}$ is 0.2 and 10 for E_w.

In practical conditional generation, a single protein condition may admit multiple valid peptide binders. We therefore perform multi-candidate stochastic decoding per query protein. Given a conditioned vector "prot_topo", we generate N (default 8) peptide candidates by

sampling from the autoregressive factorization. At each decoding step, we control diversity by sampling from a temperature-scaled next-token distribution after truncating low-probability residues, while restricting candidates to valid amino-acid tokens (including UNK when enabled) and EOS. To reduce duplicated candidates, we apply a lightweight presence penalty that down-weights tokens already appearing in the partial sequence. We finally deduplicate candidates and keep the top-N unique sequences by model score. Each generated sequence s is assigned a confidence score computed as its length-normalized log-likelihood under the same model:

$$\text{score}(s) = \frac{1}{|\ s\ |^{\alpha}} \sum_{t=1}^{|s|} \log\ p(s_t \ \mid\ s_{<t},\ \text{prot\_topo}),$$

where α is a length-normalization exponent (default $\alpha = 1$). The score provides a consistent ranking across candidates and is used for selection and downstream analysis.

Training procedure

Training is performed on paired protein–peptide examples using teacher forcing. In each minibatch of size b, we first transfer precomputed ESM protein token embeddings $\mathbf{X}^{(\text{prot})}$ and masks $\mathbf{M}^{(\text{prot})}$ for the selected protein indices from CPU to GPU. We then encode them with the protein encoder to obtain batch protein representations $\text{prot\_init} \in \mathbb{R}^{b \times D}$. Next, using the frozen dual-encoder retriever, we compute query retrieval embeddings for the batch proteins and retrieve a context set of top-K candidate peptides per protein by maximum-inner-product search against the CPU-resident peptide library, yielding corresponding indices top_idx.

We then gather the retrieved peptide token embeddings and masks for top_idx from CPU to GPU and encode them to obtain candidate peptide representations $\text{pep\_init\_sub} \in \mathbb{R}^{b \times K \times D}$. These protein and peptide embeddings are fused to a topology-conditioned protein embedding $\text{prot\_topo} \in \mathbb{R}^{b \times D}$, which serves as the conditioning vector for sequence generation.

During peptide generation training, the Transformer decoder receives prot_topo as a single memory token and produces corresponding peptide vocabulary logits. As described before, we use a primary autoregressive cross-entropy objective plus an auxiliary span-masked language modelling (Span-MLM) objective with selective bidirectional attention for masked queries. We use AdamW with learning rate 2×10^{-4}, minibatch size 16, and gradient clipping to a global norm of 1.0 for optimization.

We hold out a validation set and use it to monitor training progress. Since peptide generation is inherently multi-solution under a fixed protein condition, we design two complementary evaluation views: (i) teacher-forcing metrics that reflect likelihood modelling and optimization stability, and (ii) free-generation metrics that reflect multi-candidate conditional generation.

For teacher-forcing, we compute token-level negative log-likelihood (NLL) over all non-PAD positions:

$$\mathrm{NLL} = \frac{\sum_{n=1}^{b} \sum_{t=1}^{T} \mathbf{1}[y_{(n,t)} \neq \mathrm{PAD}] \left(-\log p\left(y_{(n,t)} \mid x_{(n,\leq t)}, \mathrm{prot\_topo}_n\right)\right)}{\sum_{n=1}^{b} \sum_{t=1}^{T} \mathbf{1}[y_{(n,t)} \neq \mathrm{PAD}]},$$

where b denotes the minibatch size, T is the padded target length (including EOS), n is sample index and t is token position. We additionally report perplexity PPL = exp (NLL) and token-level top-5 accuracy over non-PAD positions:

$$\mathrm{Acc}_{\mathrm{token}} = \frac{\sum_{n,t} \mathbf{1}[\hat{y}_{(n,t)} = y_{(n,t)}]\mathbf{1}[y_{(n,t)} \neq \mathrm{PAD}]}{\sum_{n,t} \mathbf{1}[y_{(n,t)} \neq \mathrm{PAD}]}.$$

To diagnose over-confident distributions that can lead to mode collapse in generation, we also report the average predictive entropy across non-PAD positions:

$$\mathrm{Ent} = \frac{\sum_{n,t} \mathbf{1}[y_{(n,t)} \neq \mathrm{PAD}] \left(-\sum_{v \in V} p_{(n,t)}(v) \log p_{(n,t)}(v)\right)}{\sum_{n,t} \mathbf{1}[y_{(n,t)} \neq \mathrm{PAD}]},$$

where $p_{(n,t)}(v)$ is the probability assigned to token v at position t for sample n. We also report the average target length to contextualize these token-level statistics.

For free-generation, we aggregate all associated ground-truth peptides into a multi-reference set $S^*(n)$ for each protein in the validation set. We then generate N candidate peptides per protein by stochastic autoregressive decoding conditioned on $\mathrm{prot\_topo}_n$. Each generated candidate s is assigned a confidence score given by its length-normalized log-likelihood:

$$\mathrm{score}(s) = \frac{1}{|s|^{\alpha}} \sum_{t=1}^{|s|} \log p(s_t \mid s_{<t}, \mathrm{prot\_topo}_n),$$

where $|s|$ is the generated length for peptide sequence s, $s_{<t}$ denotes the prefix before position t, and α is a length-normalization exponent used to make scores comparable across different lengths.

Ablation studies

We performed two complementary ablation analyses to evaluate the contribution of retrieved peptide context and topology-conditioned evidence integration. The first analysis was a training-level design ablation, in which each model variant was trained and evaluated under matched settings. The second analysis was a fixed-checkpoint perturbation analysis, in which the trained full BOND-PEP checkpoint was kept unchanged and only the conditioning pathway was perturbed at inference time.

For the training-level design ablation, all variants used the same retriever, retrieval library, context budget, decoder architecture, training recipe, decoding budget, and evaluation protocol unless otherwise stated. The full model ("Full") used target-relevant retrieved peptide context followed by topology-conditioned bipartite message passing. In the random-context control ("Rand"), the target-relevant retrieved peptides were replaced by randomly sampled peptide context while preserving the same number of context peptides. In the no-topology control ("–Topo"), the topology conditioner was removed, so the generator could not use the bipartite topology-conditioned alignment module. In the mean-pooling control

(“Mean”), the retrieved peptide context was retained but topology-aware bipartite message passing was replaced by simple mean pooling. These variants were used to test whether performance depended on target-relevant retrieval, topology conditioning, and topology-aware context integration during model training.

For the fixed-checkpoint perturbation analysis, we used the trained full BOND-PEP checkpoint without retraining. All perturbations were evaluated on the same strictly held-out benchmark using the original context budget, stochastic decoding, and fixed random seed. The intact full model was used as the reference condition. In the “No context” perturbation, retrieved peptide-context embeddings were zeroed while preserving the graph size. In the “Mismatched context” perturbation, the query target was kept unchanged but its retrieved peptide context was replaced by context retrieved for another held-out target. In the “Edge-shuffled topology” perturbation, the correct retrieved peptide nodes were preserved but the protein-peptide topology edges were permuted. These controls tested whether the trained full model used informative retrieved context, target-context matching, and correct topology-conditioned edge assignment at inference time.

Baseline generation settings and candidate-budget alignment

For the held-out strict benchmark, BOND-PEP generated 8 peptide candidates per target by stochastic autoregressive decoding. These 8 decoded candidates formed the final evaluated candidate set for downstream AlphaFold-Multimer structure prediction. To make the structural comparison fair across methods, PepPrCLIP, PepMLM and RFdiffusion were also evaluated using 8 retained candidates per target. PepPrCLIP was run according to its published generation procedure. For each query target, we randomly sampled 100 source peptides from the training set. For each source peptide, 1000 noised peptide candidates were generated using the reported noise-scaling range k = 5-22. This produced approximately 1×10^5 raw candidates per target. These raw candidates were then ranked by the PepPrCLIP CLIP-predicted binding score, and the top-8 candidates were retained for AlphaFold-Multimer structure evaluation. RFdiffusion was run according to its published peptide-design procedure. For each target, we generated 8 independent RFdiffusion peptide binder backbones, with the peptide length fixed to the corresponding reference PDB peptide length. Each backbone was converted into one peptide sequence using ProteinMPNN. The resulting 8 RFdiffusion peptide sequences were retained for AlphaFold-Multimer structure evaluation. PepMLM was run according to its published generation procedure as well. The target protein sequence was provided as input, followed by a designated number of mask tokens at the C terminus. The number of mask tokens was set to the corresponding reference PDB peptide length for each target. PepMLM then decoded peptide residues at the masked positions. We let PepMLM generate 8 peptide candidates per target and retained them for AlphaFold-Multimer structure evaluation. Unless otherwise specified, method-specific parameters were kept at the default settings. For all methods, each retained target-peptide pair was co-folded with the same AlphaFold-Multimer protocol, and candidate-level ipTM was defined as the highest ipTM among the final five structural outputs.

For the external panel, we first assembled the reference peptide candidate pools from the corresponding source study. These pools contained 6 PepPrCLIP peptides for β-catenin, 20 PepPrCLIP peptides for UltraID, 10 PepPrCLIP peptides for SS18-SSX1 and 4 PepMLM peptides for NCAM1. The peptide with the highest candidate-level ipTM was recorded as the reference entry. For BOND-PEP, we used the same target-specific candidate-count budget as the corresponding reference pool. Each BOND-PEP candidate was co-folded with the target

and the candidate with highest ipTM was selected and reported in Table S8. This procedure provided a fully matched comparison.
